# Addressing vulnerability, building resilience: community-based adaptation to vector-borne diseases in the context of global change

**DOI:** 10.1186/s40249-017-0375-2

**Published:** 2017-12-11

**Authors:** Kevin Louis Bardosh, Sadie Ryan, Kris Ebi, Susan Welburn, Burton Singer

**Affiliations:** 1 Department of Anthropology, University of Florida, Gainesville, USA; 2 Emerging Pathogens Institute, University of Florida, Gainesville, USA; 3 Department of Geography, University of Florida, Gainesville, USA; 4 Department of Global Health, University of Washington, Seattle, USA; 5 Centre of Infectious Disease, University of Edinburgh, Edinburgh, UK

**Keywords:** Vector-borne disease, Community participation, Social science, Adaptation, Resilience, Climate change, Global change, Global health

## Abstract

**Background:**

The threat of a rapidly changing planet – of coupled social, environmental and climatic change – pose new conceptual and practical challenges in responding to vector-borne diseases. These include non-linear and uncertain spatial-temporal change dynamics associated with climate, animals, land, water, food, settlement, conflict, ecology and human socio-cultural, economic and political-institutional systems. To date, research efforts have been dominated by disease modeling, which has provided limited practical advice to policymakers and practitioners in developing policies and programmes on the ground.

**Main body:**

In this paper, we provide an alternative biosocial perspective grounded in social science insights, drawing upon concepts of vulnerability, resilience, participation and community-based adaptation. Our analysis was informed by a realist review (provided in the Additional file 2) focused on seven major climate-sensitive vector-borne diseases: malaria, schistosomiasis, dengue, leishmaniasis, sleeping sickness, chagas disease, and rift valley fever. Here, we situate our analysis of existing community-based interventions within the context of global change processes and the wider social science literature. We identify and discuss best practices and conceptual principles that should guide future community-based efforts to mitigate human vulnerability to vector-borne diseases. We argue that more focused attention and investments are needed in meaningful public participation, appropriate technologies, the strengthening of health systems, sustainable development, wider institutional changes and attention to the social determinants of health, including the drivers of co-infection.

**Conclusion:**

In order to respond effectively to uncertain future scenarios for vector-borne disease in a changing world, more attention needs to be given to building resilient and equitable systems in the present.

**Electronic supplementary material:**

The online version of this article (doi: 10.1186/s40249-017-0375-2) contains supplementary material, which is available to authorized users.

## Multilingual abstracts

Please see Additional file 1 for translations of the abstract into the five official working languages of the United Nations.

## Background

Public health practitioners are increasingly recognizing that health, disease and wellbeing in the twenty-first century are influenced by an unprecedented number of global changes and challenges [1]. The ramifications of post-Second World War modernity – of rapid economic growth, resource exploitation and greenhouse gas emissions – have resulted in climatic and ecosystem shifts altering the thresholds of our planet. In an interconnected world, change is occurring across social, environmental and climatic scales and affecting human, animal and natural systems in irredeemably complex and yet inadequately understood ways.

Policies, research initiatives and programmes have now emerged aimed at highlighting, and addressing, the negative effects of global change on human health [2]. High-level policy advocacy has followed, including a landmark 2008 World Health Assembly resolution, Climate Change and Health (WHA61.19), and the inclusion of health in National Adaptation Plans (NAPs). The Paris Agreement, reached during the UN Climate Change Conference of the Parties (COP 21) in 2015, aims to keep global warming “well below” 2 Degrees Celsius in order to protect peoples “right to health” [3]. Regional health-related strategies in Africa, the Mediterranean, Asia and the Americas have also been developed, aimed at enhancing resilience and preparedness.

These efforts recognize the potential for global changes to influence the incidence and distribution of vector-borne diseases (VBDs), which continue to be major sources of death, disease and disability worldwide [4–7].^1^ With half of the world’s population currently at risk, a few simple statistics reveal the importance of VBDs at a global scale: an estimated 1 billion people are infected annually and more than 1 million die, accounting for 17% of the global burden from infectious diseases [4]. A range of mosquitoes, sandflies, ticks, triatomine bugs, tsetse flies, fleas, black flies, aquatic snails, and other vectors are implicated. Many are zoonotic, or are at least influenced by livestock and wildlife populations. Their health consequences and disease ecologies are diverse, making generalizations difficult. However, most disproportionately impact people in tropical climates with inadequate access to health services, poor housing, weak governance structures, and socio-economic underdevelopment. While they can cause major epidemics that impact regional economic productivity, they also enact a quieter “hidden” endemic toll on local communities, perpetuating poverty, disability, malnutrition and social isolation.

Over the last two decades, major progress has been made in tackling the burden of VBDs – for example, with malaria [8] – although progress has not been evenly distributed across the world, or across all diseases. Looking to the future, a spectrum of global challenges will influence, for better or worse, these epidemiological and programmatic trends. Understanding how this will occur, and with what consequences, needs to transcend simplistic perspectives to account for the complex interactions between hosts, pathogens, vectors, humans and environments. Emerging VBDs, such as Zika virus (ZIKV) and Chikungunya (CHIK), have recently infected millions. First identified in a rhesus monkey in 1947 along the shores of Lake Victoria, ZIKV has been linked to thousands of cases of microcephaly in Latin America and the Caribbean, and other neurological and developmental disorders in infants [9]. Other unknown infections will certainly emerge in the future; there are at least 500 known arthropod-borne viruses circulating in nature [10]. The acknowledged failings of global health institutions and actors to respond timely and effectively to such emerging diseases – shown in the recent West African Ebola epidemic – raises serious questions about the structure of global health preparedness, and the need for more community-orientated approaches [11, 12].

Significant gaps remain that pervade current policy frameworks and programme mechanisms. Most research continues to be about conceptualizing how human systems might change, or how they might need to change, to future scenarios, generating a “wish list” of policy entry points – as seen in Intergovernmental Panel on Climate Change (IPCC) reports and National Adaptation Plans (NAPs) [13]. There is a sort of paralysis in regards to effective and feasible action. Campbell-Lendrum et al. [7] have argued that there is a need to better define the kinds of VBD control decisions needed, in what contexts, their time period(s) and what variables need to be accounted for. The predominate focus tends to remain on climate change (and not the broader emphasis on global change) and remains largely ‘siloed’ in particular sectors and disciplines, rather than taking a more cross-sectoral and holistic approach [5, 14, 15]. Different stakeholders have differential perspectives about what is most important and how policies and programmes should be designed and implemented (for an example, see Wei et al.’s [16] study among different tiers of Center for Disease Control and Prevention [CDC] staff in China).

Where VBD adaptation policies currently exist, they are ad hoc and fragmentary, with significant gaps in detailing how such policies are to be funded, translated into practice and evaluated (see Table 1). There also tends to be an over-emphasis on the importance of technology and biomedical expertise, while the needs and capacities of vulnerable population groups and local stakeholders are absent, despite the rhetoric of social justice that pervades the climate change literature. Major thematic areas that are underdeveloped for VBDs include: the importance of community participation and citizen engagement, the role of social differentiation and the links between disease and wider system dynamics, all of which has long been promoted in global health as an integral component of VBD control [12]. In order to guide funding and prioritization efforts, and to realign the agenda, this paper explores the relevance of key concepts of vulnerability, resilience, adaption and community-based approaches.

**Table 1:** Evaluation of vector-borne disease in national adaptation plans across 6 Mediterranean countries

A recent evaluation explored adaptation plans in Spain, Italy, Malta, Turkey, Israel and Egypt regarding climate change and vector-borne diseases. As wealthier countries, they are likely to reflect more progressive policies than most least developed countries (LDCs) and low- and middle-income countries (LMICs), and focused on early warning systems, response plans and training. The authors found substantial variation in the actual details provided, and questioned some of the omissions given the needs of the countries involved. For example, Turkey emphasized the vulnerability of seasonal agricultural workers, but other countries did not identify sub-groups at higher risks of infection. Although cross-border movement is important for many countries, only Israel emphasized surveillance and monitoring of border areas and the need to improve vector management regulations for local authorities. Two other important weaknesses were also identified. First, most countries did not detail the agencies that would be responsible for implementation, or discuss mechanisms for collaboration and funding. Second, there was little attention given to education, and a complete lack of discussion of community participation and public engagement in policymaking and implementation. These findings echo an earlier study in 14 OECD countries on infectious disease and climate change adaptation (see [84]). From Negev et al. [5]	

In order to guide our analysis, we conducted a realist review [17] on community-based interventions for VBDs with the goal of relating past approaches and lessons learnt to the context of future global change (see Additional file 2 for our analysis of this material). We explored seven major VBDs that are of significant public health importance and show sensitivity to social, environmental, and climatic change (See Table 2): malaria, schistosomiasis, dengue, Chagas disease, human African trypanosomiasis (HAT), leishmaniasis and Rift Valley fever (RVF). This methodological approach allowed us to develop a panoptic perspective on the types of approaches that are available and have been tested and evaluated for these 7 VBDs. In our review, we asked: *what works, why, in what context and for whom?* Seven major types of community-based activities were identified, explored and analysed, results of which are presented in the Additional file 2 to this paper, with specific examples provided from a country-level. As summarized in Table 3, this included: 1) Vector surveillance and risk mapping; 2) Housing and the domestic environment; 3) Modifying natural environments; 4) Animal-based interventions; 5) Water, sanitation and hygiene (WASH); 6) Chemical vector control; and 7) Access to biomedical interventions.

**Table 2:** Summary of the 7 VBDs in this review

Disease	Vector	Current population affected	Current WHO control/elimination targets
Chagas disease	Triatomine bugs	6 to 7 million, in the Americas	Elimination of peridomiciliary infestation by 2020
Dengue	*Aedes* mosquitoes	390 million annually. At least 500 000 develop severe dengue, and 2.5% of these die	Reduced rates of morbidity by at least 25% and of mortality by 50% by 2020
Human African trypanosomiasis	Tsetse flies	Estimate of 20 000 new cases (2014), in Africa	Elimination in 80% of foci by 2015 and total elimination by 2020
Leishmaniasis	Phlebotomine sandflies	Over 1.5 million cases, 90% of which are in India, Bangladesh, Sudan, South Sudan, Ethiopia and Brazil	100% case detection and treatment of visceral leishmaniasis in South Asia by 2020
Malaria	*Anopheles* mosquitoes	214 million cases in 2014 and 438 000 deaths	Reduced incidence and mortality by at least 90% by 2030
Rift Valley Fever	Several mosquito species	Unclear; epidemic-prone zoonotic disease	Unclear
Schistosomiasis	Freshwater snails	Over 200 million people in 2013	Elimination as a public health problem globally by 2025

**Table 3:** Identified community-based interventions in the review

Intervention domain	Examples discussed in the realist review
Vector surveillance and risk mapping	• Health and Demographic Surveillance Systems (HDSSs) • Community-based vector surveillance • Community-based clinical case surveillance
Housing and the domestic environment	• Bednet distribution and promotion • Larval source reduction campaign, and garbage collection • Housing improvement
Modifying natural environments	• Cleaning drains • Clearing vegetation from waterways • Promoting changes in agricultural practices
Animal-based interventions	• Strengthening veterinary services • Zooprophylaxis • Insecticide-treated dog collars • Animal movement restrictions
Water, sanitation and hygiene	• Latrine and sewage improvement • Changes in water use and supply • Hygiene health promotion
Chemical vector control	• Combined use of IRS with health promotion • Community-directed larviciding
Access to biomedical interventions	• Village-level drug treatment systems • Mass drug administration

Here, we situate our analysis of the existing community-based VBD intervention literature within the context of global change processes, the broader socio-ecological systems theory literature, social science knowledge and concepts of vulnerability and adaptation. Our paper is divided into three sections. Section 1 outlines how different global change processes are predicted to impact VBDs, and discusses the complexities and uncertainties involved and the importance of a biosocial perspective. Section 2 introduces key concepts from the social science literature on vulnerability, resilience, participation and community-based adaptation. Section 3 provides a synthesis and critique of best practices for community-based approaches to guide vector-borne disease strategies in the context of global change. Additional information is provided in the Additional file 2, including the methodology and results of the literature review that informed this paper.

## Main text

### Section 1: Global change and vector-borne disease: a biosocial perspective

In this background section, we present a biosocial perspective on global change processes – major social, environmental and climatic change – and discuss some of the predicted impacts on vectors/pathogens, the epistemological challenges involved in knowing about these dynamics and the implications of this for policy and practice.

#### Climate change and variability

With an upward estimate of a 4-5 degree Celsius increase this century, increased vector densities and geographical spread into previously cooler, temperate regions is very likely for many VBDs [18–20]. Vector and parasite development tends to occur more rapidly at higher temperatures. Climate change has been associated with reduced vector mortality rates, a shift from seasonal to perennial transmission, and epidemic events due to extreme climate events, like flooding [21, 22]. In other places, however, hotter and drier conditions will reduce vector populations, such as tsetse flies, and drive reductions in disease incidence [23].

Most VBDs can be transmitted by multiple species of vectors (*Schistosoma mansoni* is spread by 30 freshwater snail species and human African trypanosomiasis [HAT] by over 20 varieties of tsetse fly). While certain species certainly maintain a dominant role in disease ecology, vector distributions will be shaped by the ways that temperature and precipitation differentially influence vector physiology, preference for different hosts and biting rates [24, 25]. Vector populations are influenced by rainfall, temperature and humidity that, in turn, influence land cover and land use. Stensgaard et al. [26] predicted significant decreases of *S. mansoni* in west and central Africa by 2080, with increases in eastern and southern Africa. Studies on malaria distribution have projected increased transmission at higher altitudes – in the highlands of Africa, parts of Latin America and Southeast Asia [27, 28]. More recent studies show increases in areas other than the highlands, depending on demographic, socio-economic and ecological factors [21]. Most studies reveal changes at the margin of current distributions, where non-immune populations present “endemically unstable areas”, which will shift patterns of endemic/epidemic conditions [19, 21].

#### Land use, biodiversity and agricultural change

Ecosystem disturbances to land and biodiversity will also influence VBDs. This includes global trends in deforestation, soil erosion, desertification, wetland degradation, and species extinctions [29]. Over 2.3 million square kilometers of primary forest have been cut down since 2000, and one in ten animals and plants are estimated to be extinct by 2050 [30, 31]. With a human population of 9.6 billion projected for 2050, new pressures are being put on natural resources, challenging current industrial and small-scale agricultural systems.

Deforestation is one of the most conspicuous anthropogenic changes. Cutting down forest creates new spatial interfaces that promote increased contact between biting insect vectors, their animal hosts and humans. Threatened forests within areas of malaria risk cover over 100 million people and approximately 5 million square kilometers in the Amazon region, Central Africa, Western Pacific, and South-east Asia [32]. Impacts are variable depending on the specific ecological niche of the vector species; forest clearances may create favorable conditions for the proliferation of heliophilic malaria vectors like *Anopheles gambiae* in Africa but reduce Anopheles dirus in Southeast Asia that prefer forest cover [20]. More complex transformations of malaria transmission, as exemplified by frontier malaria in the Brazilian Amazon, are consequential to forest clearances that expand and change land utilization patterns [33]. Forest clearances have been responsible for multiple epidemics of leishmaniasis, Chagas disease and trypanosomiasis due to logging, road construction, fires and new human settlements.

By influencing land cultivation and vegetation type and cover, new agricultural production dynamics shift vector-animal relations. The example of trypanosomiasis is instructive. Agricultural change can be protective, as when farmers clear tsetse-infested forests and swamps and apply pyrethroid-based pesticides on crops and livestock [34]. But it can also reduce biodiversity and the range of reservoir hosts that tsetse feed on, driving greater infection in livestock and increased human transmission close to homesteads. In this sense, biodiversity can act in a protective function against increased trypanosomiasis infection. Infringing on forest ecosystems, cutting down trees, planting crops, raising domestic livestock, constructing settlements, and hunting wild animals all affect vector-feeding patterns. While changing land use and agricultural patterns may ultimately reduce vector-borne infections – as hypothesized for the history of malaria in Europe and elsewhere [35] – transitional periods present heightened risk due to new contact interfaces.

#### Dams, irrigation and water

Water and sanitation are implicated in vector breeding as well as human behavioral practices that facilitate VBD transmission. More than 40% of the world population (2.6 billion) has inadequate access to improved sanitation, with many continuing to defecate in open areas, and nearly 1.1 billion lacking access to improved drinking water [36].

Global change will stretch across three main water-related domains in relation to VBD [20]. First, water shortages and the demand for electrification and economic development will drive increased numbers of man-made lakes, dams and polluted waterways. These affect ecosystems, societies and political economies, with numerous examples of water developments driving the impoverishment of people and spread of VBD [37]. The environmental effects of small and large dams are responsible for an estimated 1.1 million cases of malaria each year in Africa [38]. Numerous examples exist of dams contributing to epidemics of schistosomiasis by altering the habitat of snail populations – in Lake Volta in Ghana and Lake Nasser in Egypt. Changes in canals, lack of drainage for sewage and poor sanitation can also drive increased infection [39].

Secondly, irrigation schemes and other agricultural practices are predicted to change surface flooding and soil saturation for larvae and snails. Rice is grown in flooded paddies that are perfect breeding sites for *Anopheles gambiae*, the principal vector of malaria in Africa. Larval densities are related to these agricultural practices, when stagnant water pools accumulate in ditches and fallow fields. But in many areas of Africa, irrigation can actually reduce malaria rates by driving economic development and increasing less vector competent malarial mosquitoes, which has been dubbed the ‘paddies paradox’ [40].

Lastly, water shortages will drive changes in water usage patterns and behaviors [41]. Codjoe and Larbi [42] explored public perceptions of climate change and schistosomiasis in Ghana, and found that community members perceived that warmer temperatures were encouraging greater contact activity with snail-infested waters, such as swimming, washing and bathing. Warmer climates will likely drive households to maintain more water containers around their homes for storage, potentially contributing to more breeding sites for *Aedes* mosquitoes. Cattle herders may be driven to cluster in closer proximity to fewer waterholes, sharing them with wildlife, tsetse flies and other vectors [43].

#### Urbanization and economic development

Processes of urbanization and economic development are equally important determinants of VBD epidemiology. The rate of urbanization has accelerated dramatically, with 60% of the world population predicted to live in cities by 2030, making modern cities one of the dominant ecosystems on earth. Unplanned urbanization, including the proliferation of slums that lack safe drinking water, drainage systems, and garbage collection, will increase [44, 45].

Greater house infestations of triatomine bugs, that spread Chagas disease, can occur from simple improvements in public streetlights, as documented in Yucatan, Mexico [46]. Electric pumps installed as part of a rural electrification project in Brazil were found to be a risk factor for schistosomiasis spread [47]. The urban mosquitos *Aedes aegypti* and *Aedes albopictus* that spread dengue fever, Zika, and chikungunya proliferate in man-made containers, such as tyres, pots and water storage containers [48]. These vectors proliferate in long occupied urban spaces where impoverished people live in crowded conditions; major epidemics of dengue continue to occur in the Favelas of Latin America [49].

The exact ways in which urbanization and vector densities interact vary. Levy et al. [45] explored changes in the built environment of a major city in Peru, and found that Chagas disease (influenced by housing quality) was most prevalent among older (and slightly more wealthy) sections of the city due to land tenure security, which drove residents to invest more in their housing and perpetuate Triatoma infestans colonies. Visceral leishmaniasis (VL), generally a rural zoonotic disease, has now spread to urban centers in Brazil through rural-urban human migration [50]. In contrast, models of malaria and dengue that account for climate and economic development have shown general decreases in distribution by 2050 due to increased socio-economic development trends, including better housing, piped water access, air conditioning, improved health outreach services and other factors [51–53]. This optimistic outlook may hide differential economic prosperity; whether such changes will occur among urban slum communities remains unclear.

#### Population movement and conflict

Migration, population growth and conflict will also continue to affect VBD [54]. In a world of flux, people, goods and vehicles are constantly in motion across borders and seas. Trade and travel translocate vectors and pathogens to new areas. According to the United Nations High Commission for Refugees, current geopolitical conflicts (in Syria, Yemen, Afghanistan, Iraq, South Sudan, Nigeria and elsewhere) have caused the highest number of internally displaced persons and refugees since the Second World War, estimated at 60 million. This number is unlikely to be reduced soon, as climate change (floods, storms, landslides, and sea level rises), desertification and population growth maintain insecurity and drive migration of so-called ‘climate refugees’ [55].

Natural disasters and armed conflict disrupt existing medical services and outreach, while also contributing to landscape changes [56]. In Uganda, cattle restocking after decades of military conflict drove an epidemic of sleeping sickness in disease-free areas [57]. Outbreaks of cutaneous leishmaniasis have been reported in conflict regions of Afghanistan, among both civilians and army personnel [58].

Human movement between low- and high-risk areas is also important. In the Greater Mekong sub-Region, where artemisinin-resistant malaria has emerged, an extensive system of dams, planned in the region, are predicted to drive future migrations that will perpetuate malaria transmission [59]. Migration has been implicated in the spread of Chagas disease from Latin America into the United States, Spain and other nations [60]. Religious pilgrims have spread leishmaniasis [61], while trade routes for domestic livestock have helped transmit RVF between the Horn of Africa and the Middle East [62]; the explosive spread of Chikungunya and Zika were both facilitated by the modern aviation network [9].

#### Biological change and drug resistance

VBD control efforts implemented at scale, such as insecticide-treated bednets (ITNs), can change the ecology/biological of vectors and pathogens. A major threat to global malaria control efforts, for example, is pyrethroid resistance in African anopheline mosquitoes and artemisinin-resistance [63]. The former may be driven by large-scale use of agrochemicals [64]. Resistance is also a key issue for drug treatments for HAT, leishmaniasis and possibly schistosomiasis.

However, biological change can also be protective. A study on the Kenyan coast found that malaria vectors and transmission had changed substantially over 20 years [65]. In the context of expanding irrigation, economic development and wide-scale distribution of ITNs, a shift from human to animal feeding (zooprophylaxis) and a general reduction in *Anopheles* densities occurred, and reduced malaria burdens in people.

#### Social and political change

Lastly, VBDs are also influenced by the context of social, cultural and political change, which have major effects on the social determinants of health, mediating financial flows and human resources and shaping the delivery of healthcare services and disease prevention initiatives [66]. These include decentralization and liberalization in civil service reforms. Balen et al. [67] drew attention to the ways in which changes in medical insurance served as a major barrier to schistosomiasis treatment among the poor in China. This is an illustration of how healthcare will be affected by shifting patterns of access, treatment, provision and health seeking behavior, which will also be influenced by changing cultural norms and values.

Public policy changes are paramount to the structure of these services. International funding and national budgets play a substantial role, and any increase or contraction of funding will have dramatic downstream effects. In important respects, dependence on foreign aid and outside experts in shaping the public health agenda can be antithetical to country-level ownership and sustainability. These trajectories are among the hardest to anticipate, making our understanding of their impact of great importance.

#### Interconnectivities and methodological issues

All of these global changes – climate change, land use, agriculture, dams, irrigation, urbanization, economic development, population movement, conflict, socio-political shifts, biological change, drug resistance, etc. – do not occur in isolation, or in a vacuum (see Table 4). They often occur in tandem, and in complex dynamics across overlapping scales where they generate significant feedback loops with multiple degrees of impact [68].

**Table 4:** Malaria in India

Nearly 14% of the Indian population is at high-risk for malaria, with over 1 million infections and 2000 deaths each year. Climate change is predicted to shift the malarial zones in India. However other factors are likely to exert an equal, if not more, significant effect. This includes economic growth, irrigation and farming, urbanization, deforestation in tribal areas, and improved primary healthcare to indigenous groups. These will not only shift vector and pathogen dynamics, but also the health status of at risk populations, impacting anemia, poverty, illiteracy, immunity and nutrition, which also influence the likelihood of adverse clinical disease. Development activities, from dams, canals and road and railway construction will also influence seasonal malaria trends. The expansion of malaria into highland areas will be influenced not only by climate, but also rapid population growth and deforestation, especially around valley ecosystems. From Garg et al. [139]	

The recognition that global change is complex and that future disease scenarios are uncertain brings with it major methodological challenges [69–72]. Models are imperfect, and can rarely account for all the cross-scale interactions and feedback loops. The quality and quantity of data is often simply lacking or inadequate to generate meaningful parameters. Hence our understanding of current and future interconnectivities is limited by our science and our ability to project and comprehend future trends. We model for insight, and need to be reflective both of the strengths and limitations of these models.

The more pessimistic view is that the current nexus of global change will prelude effective adaptation and mitigation, and that increased vulnerability, infection and epidemics will be inevitable [19]. However predictions of expanded transmission should be placed in parallel to current control initiatives [73], economic development trends [51–53] and future adaptations undertaken by local populations and public health agencies. Most current epidemiological models tend to neglect how local communities adapt in the context of an epidemic, or how they use socio-cultural capital to mitigate endemic disease challenges [74]. Furthermore, the technology of ecosystem surveillance, of understanding the complex relationships and feedback loops of change, is undergoing rapid change, with capabilities at multiple scales improving dramatically [75, 76].

### Section 2: Promoting adaptation: communities, capacities and change

Tackling VBDs in the context of global change requires an appreciation of existing vulnerabilities and how to address them. Sutherst [20] proposed a generalized risk analysis framework, where vulnerability is viewed as the level of exposure and sensitivity to a VBD minus the adaptive capacity of populations and systems to adjust to them. In simpler terms, vulnerability can be viewed as the ‘capacity to be wounded’ by change or the ability for a natural or social system to be ‘prone to damage’ [77].

The concept of ‘adaptation’ is also important [78]. With roots in the natural and social sciences, particularly evolutionary biology and cultural anthropology, the term has often been used to denote genetic or behavioural changes, as well as changes in cultural practices and beliefs, that assist organisms (humans) to survive and reproduce, all in the context of environmental change and hazards [79]. The capacity to adapt is closely related to other concepts in ecological systems theory, such as coping, flexibility and resilience. These terms broadly refer to the ability for a system to undergo stress and change, while maintaining its essential function(s) (although there is significant debate within the climate change community about whether returning to a system’s original state is even desirable in the context of changing weather patterns).

Poverty enhances vulnerability to VBDs in multiple ways, mainly by removing the capacity for people to cope with and address health risks [80]. Different temporal and spatial scales are at play, and extend across a diverse number of social, cultural, political, economic, environmental, climatic, and biological determinants. Such multi-layered relationships have been likened to “Russian dolls” where concentric relationships are interrelated, and outer layers either hinder or facilitate the resilience of spaces within the inner layers [77]. A number of recent spatial models have attempted to map social vulnerability to VBD, such as malaria in East Africa and dengue in Latin America, and have confirmed this perspective [81, 82]. Studies on malaria in Rwanda and Tanzania, for example, found that population change, droughts and famines, irrigation, lack of bed net ownership, and poor housing material were significantly correlated with an increased risk of malaria [83, 84]. Whether these risks are truly nested or simply interrelated at multiple scales, they present a complex network of factors to address.

Vulnerability is not easily measurable and translatable across contexts, and efforts to standardize metrics for decision-making risks generating vast simplification [85]. A more contextualized approach is required. Populations most at risk from VBDs tend to depend heavily on natural resources, the informal economy, and occupy areas prone to shocks, have inadequate access to social services and have limited capacities to cope and adapt. Poverty traps rest in the breadth of choices needed for adaptation. Different social groups are vulnerable in different ways, influenced by place of residence, ethnicity, social class, gender, occupation, religion, and age. For instance, men suffer increased risk of VBDs based on occupation in extra-domestic habitats (as farmers, bee-keepers, charcoal producers) while women may have increased risk based on their housekeeping roles in the domestic habitat (where vector densities are highest and stable year-round) [86]. Typically, women, the elderly, children, the disabled and indigenous populations and minorities tend to experience the highest degree of socio-economic marginalization, and are therefore most vulnerable to shifting conditions [87, 88].

This is what is commonly meant by disease causing a “cycle of poverty.” Low socio-economic status tends to translate into limited political access, as key resources and opportunities are not accessible to the poor. This influences systemic vulnerabilities that pervade the public health system, including the lack of effective surveillance, early warning systems, equitable health governance and access to diagnosis, treatment and prevention. Geography, environment and culture also matter, as remoteness reduces access to social services, land rights maintain economic exclusions and socio-economic conditions and cultural normsdictate how people use, and who can use, health technologies. Livelihoods are influenced by access to natural, human, social, and financial resources and assets, such as soil conditions, forest resources, access to markets, social safety nets, education, political power and technologies [89]. This influences the range of VBD prevention and control tools that people use, from housing, the ability to repair mosquito nets, access to vector control team and health treatment affordability, to name a few. By understanding these relationships and social spaces, we can identify, and therefore engage with, areas for adaptation and places where capacities need to be strengthened and addressed.

Identifying vulnerabilities can help open-up policy pathways to address them by building the adaptive capacity of people, organizations and institutions. Increasing the potential for adaptation is often dependent on complex social and cognitive dynamics, such as the ability for people to learn and analyze, put learning to use, to be flexible to circumstance and to have the capacity to consider alternatives. For example, studies among small-scale farmers in Africa and pastoralists in Central Asia have found that resilience is equated to livelihood diversification, community ownership of natural resources, inter-community equity, the ability to influence policies and resources and the capacity to organize and learn new things [77, 90]. Adaptation cannot be built solely by developing sound policy, but must also appreciate how local people attempt to address problems and solicit support [91]. Constraints to human agency, and how social ecology is influenced by, and influences, structural conditions of inequality need to be accounted for and considered [92]. These are situated processes that require situated knowledge to understand, but perhaps more importantly, situated policy and programme engagement.

For these reasons, global policy debates appear to be paying greater attention to the benefits of a community-led approach in responding to global change. However in important respects, this is nothing new – sometimes it appears that a new vocabulary is simply reiterating old ideas that have long occupied ground in public health, environmental and sustainability discourses. This provokes critical questions regarding the relationships between the rhetoric and translation into practice. Nonetheless, within the climate change community itself, this is a relatively new focus, and an important one. This has become known as ‘community-based adaptation’, a concept that has become increasingly mainstreamed over the last 10 years [93–95]. This approach orientates research and intervention on the priorities, needs, and capacities of communities themselves and aims to empower local people to prepare and navigate future change [94]. It has grown from a concept and a few pilot studies to an emerging field of academic interest and NGO programmes, one that is grounded in a multidisciplinary and cross-sectoral approach.

As defined in this emerging literature, community-based adaptation (CBA) refers to the acquisition of local skills and capacities that strengthen community action to reduce climate change related vulnerability [95]. A CBA approach aims to foster effective organization of local people to participate in decision-making. It embodies small-scale, place-based analysis and action – often grassroots-driven and community-based [94]. It is more about process than outcomes. CBA also promotes linking different disciplines together – meteorologists, conservationists, biologists, climate scientists, social scientists, and others – and the forging of partnerships with communities, valuing local knowledge and having an integrated problem solving approach. To date, most efforts have involved promoting relatively small changes in livelihood patterns and local natural resource management, often in rural areas [94, 95]. This ranges from modifying water conservation strategies, diversifying incomes, enacting flood or hurricane warning systems and enhancing land management alternatives.

In many ways, CBA parallels other participatory approaches that have emerged in natural resource conservation, sustainable development and public health over the last 40, or more, years. However the focus on changing social-environmental-climatic conditions is unique, and provides an important counterbalance to the current global change debate by putting local people at the center of analysis and action. As pilot projects have proliferated, lessons are also now beginning to emerge. Key issues discussed by Ensor, Berger and Huq [93] include:

1. The problem of scale (most CBA projects are small-scale);
2. The politics of technology (how institutions and interests shape how science and technology are prioritized);
3. The lack of integrating strong and holistic ecosystem perspectives; and
4. The challenges in fostering sustainable transformations in the absence of addressing overarching socio-economic structural conditions

These challenges parallel those that continue to be voiced about the inclusion of community participation in global health. As part of the social medicine movement, since the Alma Ata Declaration (1978) and the Ottawa Charter for Health Promotion (1986), community participation and health systems strengthening has occupied an important area in global policy, albeit one that has ebbed and flowed over time [96]. While the word ‘adaptation’ is not explicitly used, there are numerous parallels to learn from and incorporate including: community uptake, ownership, equity, accountability, local empowerment and sustainability [97]. But while the benefits of participatory approaches are continuously extolled, there does continue to be relative policy neglect for their large-scale implementation, including for VBDs.

Two institutional barriers are pervasive in this regard. First, there appears to be an entrenched unwillingness for the mainstream medical establishment to move into these areas in any concerted fashion. Certainly examples do exist, but on the whole biomedicine remains removed from the social medicine movement. Second is the issue of scale. Most community-based projects remain localized, either as demonstration projects to generate research and validate an approach or due to the human capacity constrains needed to effectively run such programmes. They demand time, new skill sets, iterative learning, and the transfer of decision-making power from experts to other stakeholders, including communities. Once such approaches are brought to scale, however, there is a real danger that they become diluted as they run-up against broader bureaucratic cultures and centralized management systems. Hence it is unclear at what scale community-based approaches can be successful deployed, and the ingredients needed for effective scale-up.

If a community-based approach is going to be effectively used at large scales to help communities and health systems adapt to changes in vector-borne disease distribution and incidence, there is a need to answer some key questions: In a changing world, how can vulnerability best be addressed for VBDs? What forms of expertise are needed? How can community involvement and participation be strengthened, and in what ways and by who? What types of interventions and initiatives work best, and on what scale? And how can research, policy and practice be developed in ways that fosters accountability and equity perspectives? The following section aims to answer some of these difficult questions.

### Section 3: Research, policy and practice for community-based adaptation to VBDs

Drawing on our realist review analysis (see Additional file 2), here we highlight nine major crosscutting themes that are particularly important for VBD research, policy and practice efforts seeking to build the adaptive capacity and resilience of local communities to address VBDs.

#### The problem of uncertainty

Addressing the effects of global change on VBD will require difficult policy decisions about what types of activities to fund and prioritize in the context of resource limitations, complexity and uncertain futures. Understanding future vector dynamics and disease ecology is imperative to determine important trends and patterns. Greater focus on collecting detailed local- and national-level entomological, incidence and prevalence data are certainly needed, and require more sustained investment. Without this data, it is difficult to target high-risk geographical areas for community-based interventions.

One of the most effective ways to protect populations from future threats is to continue and expand current efforts. If many of the WHO targets for the control of VBDs, such as malaria, schistosomiasis, sleeping sickness, leishmaniasis, dengue and Chagas disease, are met or even advanced, there will be a much-reduced risk from other vector illnesses across the globe, and the systems and capacities put into place should have many secondary positive effects. One of the only community-based studies identified in our review that looked at local perspectives of ways to mitigate the effects of climate change on a VBD had community members in Ghana simply re-emphasize an intensification of currently accepted control approaches for schistosomiasis [42]. A second community-based study in Tanzania emphasized the need to better link livelihoods, food security and malaria control as they are impacted by climate change. This included the need to scale-up and extend current approaches, and foster collaboration between the agriculture and health sectors [98].

Research on climate change adaptation has found that policymakers find it exceedingly difficult to grapple with the long-term nature of climate change on health in resource-limited settings [99]. Citizens also find it difficult to plan for future scenarios – from climate, social change or environmental shifts – in the absence of an emergency, or visible threat. The costs of adaptation are immediately felt, but the benefits accrue with time. The mantra seems to be: what we see is what we know, and is what we will prioritize. This makes it challenging to incentivize effective adaptation policies that are not directly applicable to current priorities and interests. This realization should challenge us to identify policy spaces where planning for future global change threats can be integrated.

#### Perspective matters

This is not to say that institutional processes, policy and programme operations should stay the same – far from it. An ideal situation is where the emerging emphasis on the consequences of global change facilitates greater emphasis on using a systems approach, one that considers prevention and control initiatives in a context of flux and interconnectivity with other social-ecological problems. In short, perspectives matter – the ways in which we view problems frame the types of solutions that come to be prioritized [100].

An undercurrent of research and programmes on VBD emphasize the importance of a trans-disciplinary perspective in opening up new viewpoints and problem solving skills to address emerging challenges (see Table 5). In important ways, conventional ‘risk’ focused control approaches, aimed at stability, are unable to cope with the high level of uncertainty involved. In practice, there is much uncertainty, ambiguity and even ignorance about epidemiological trends and the impact of specific prevention or control efforts. This is enhanced when considering future possibilities. Embracing uncertainty, therefore, demands an acceptance of alternative pathways of planning and response that engage with ambiguity and ignorance [100]. Integration of multiple types of modeling and methods can enhance linkages between research and appropriate policy.

**Table 5:** Trans-disciplinary research on landscape ecology and Chagas disease in Mexico

Understanding the disease ecology of VBDs where rapid social, environmental and climatic change is occurring simultaneously is a major component of future efforts required to better design interventions. This would benefit from a trans-disciplinary approach. Most transmission ecology studies for Chagas disease are conducted by epidemiologists or entomologists, and assume that vector transmission occurs in domestic spaces. Few investigate the potential interactions between people and triatomine bugs in different landscape fragments, or incorporate social science expertise. This is especially relevant as historical mass insecticide spraying to control *Triatoma infestans* and *Rhodnius prolixus* is not effective against other triatomine species, which are zoonotic. An anthropological study was conducted in collaboration with a quantitative landscape ecology analysis on the eco-bio-social dynamics involved in *T. cruzi* presence and spread in Mexico. This included exploring the interrelationships between exposure patterns, gender, seasonality, livelihoods, local perceptions, care-seeking, and ethno-ecology. An integration of methods was used to account for spatial and temporal aspects of the parasite and disease ecology. This showed that landscapes were fragmented, and that remnant patches presented different types of risks and exposures. The study emphasized that social representations and practices of people should be viewed as part of geographical, cultural and economic heterogeneous landscapes, rather than assuming homogeneity. The results between the two disciplinary teams also validated each other, suggesting that the same integrated risk analysis framework could be extended to other communities. The vulnerability assessment provided a key step forward towards designing effective control approaches for non-domestic triatomine infestations in the study location. From Valdez-Tah et al. [86]	

Incorporating a social science perspective, one that opens up space for multiple perspectives, is essential to advance societal adaptation to VBDs in the context of global change. In some important respects, this is nothing new. There have been many reviews on the role of social science research on VBDs: for malaria [101], schistosomiasis [102] and Chagas disease [103]. There is now a sizable body of knowledge that clearly highlights the importance of fostering community participation, considering social difference, understanding complex human-animal-environment interactions and designing interventions in ways that take into account important socio-cultural and institutional dynamics.

But the translation of this knowledge into better policy and programmes is far from linear. Systems that enable the generation and utilization of social science intelligence – from the disciplines of anthropology, sociology, political science, geography, public policy, behavioral science and others, are vastly inadequate and fragmented. As shown in our review (see Additional file 2), examples do exist. But outside what are often small-scale academic research projects, these skills are not easily and consistently available for use by programme workers, practitioners and country-level managers, who are widely responsible for implementation.

Multidisciplinary research in global health has become increasingly popular under the “One Health” and “EcoHealth” movements [11]. Focused field studies that integrate social, biomedical and ecological perspectives are important, but need to be integrated with the community of policymakers and practitioners working on the ground to have the most impact [104]. Otherwise we risk becoming a “talking shop”, guilty of repackaging ideas with limited scope for actual change. Shifting conceptual trends in the current policy landscape is about attitudes, norms and values – of scientists and policymakers – and cannot happen overnight. One important pathway is to invest in nodes of change with proven histories in local contexts – centres of excellence in developing countries with a track-record of effective community-based research and policy engagement. A good example for VBDs is the Ifakara Health Institute in the Kilombero Valley of Tanzania, which has maintained stable financing, independent of fluctuating economic conditions, and generated much ground-breaking research while also having significant impact on population health [105]. Another, although perhaps less discussed pathway, is to build bridges between academia, public health agencies and the private sector in order to draw on shared concerns and promote opportunities to work together.^2^

#### Reframing surveillance – The problem of co-infection

Surveillance systems need to move beyond current disease silos to address the problem of co-infection. Current surveillance on morbidity and mortality reporting is almost exclusively focused on single diseases at a time [106]. This is despite the fact that this often does not reflect disease burden at a community level, where multiple infections cluster together in the same community and in the same individuals, mostly the very poor [107, 108]. Among vector-borne diseases, the same species of mosquito is often transmitting multiple parasitic or viral diseases in overlapping locations. Well-known examples of this are anopheles vectors that transmit both malaria and lymphatic filariasis (LF) [109] and Aedes vectors that can transmit all four of yellow fever, dengue, chikungunya, and zika viruses. Triatomine bugs that transmit chagas disease and sand flies that transmit leishmaniasis are also simultaneously present, for example, in communities in northwest Argentina [33].

Compartmentalized disease surveillance and reporting systems are accompanied by balkanized international networks of donor agencies and international organizations that concentrate fundraising, and even support research, on a single disease at a time (e.g. malaria, LF, HIV, schistosomiasis) – such projects frequently become “islands of success” in a sea of inadequate access to even the most basic healthcare services. Even the broad category of neglected tropical diseases (NTDs) pays minimal attention to the pervasive co-infection that is revealed by the limited array of community studies clearly showing this phenomenon [108].

From the perspective of understanding immunology of infectious disease, there is a growing literature documenting the complex interactive responses of distinct pathogens in a common host [110]. Failure to take account of such interactions, whether they have negative or even positive consequences for the host, can lead to inadequate clinical care, incorrect assessment of the burden of disease at the community, district, and national levels, and erroneous epidemiological projections based on mathematical modeling of disease transmission. Furthermore, the emphasis of both malaria and lymphatic filariasis control – considered separately – is currently focused on diagnosis and pharmacological treatment of infected human cases. If consideration were being given to the fact that the same vectors are transmitting both diseases, frequently in the same places, much more attention would be given to integrated vector management – an approach that remains relatively marginal in current global policy circles [111, 112]. Furthermore, from the perspective of climate change projections, the more intricate ecosystem structures that are needed to describe risk of multiple VBDs are not appropriately tuned to realities that are likely to manifest themselves in the future. It is important that we base our understanding of disease patterns, and disease prioritization, on local epidemiological dynamics; Health and Demographic Surveillance Systems (HDSSs) show great promise to provide such actionable intelligence (Table 6).

**Table 6:** The potential for health and demographic surveillance systems

A promising start at surveillance and monitoring of disease status at the community level derives from the increasing proliferation of Health and Demographic Surveillance System (HDSS) longitudinal data – see examples from The Gambia [140] and Tanzania [141]. To-date these systems report on the marginal distribution of diseases across multiple communities, making them a useful basis for district level health planning. They do not, but easily could, include reporting on co-infection and, more generally, co-morbidity. This is not an instance where new data collection is required. It is only an instance of carrying out more elaborate reporting of information already available. There are, of course, limitations on the level of detail that can be recovered from extant HDSS data due to the lack of extensive laboratory assessment of stool and blood samples as, for instance in a polyparasitism study in Cote d’Ivoire where up to 10 distinct intestinal parasites were identified in a single individual [108]. Nevertheless, reporting of co-infection as observed in extant HDSS systems would represent an important first step toward routinizing district level data that could be brought in for planning of more comprehensive control strategies tuned to the real needs of communities. This would also provide an important information base for ascertaining the extent to which very labor, and financially intensive, disease elimination programmes (e.g. malaria elimination) are worth the investment where a host of other conditions are likely to deserve much greater attention.	

In addition, the contemporary heavy focus on pharmacological interventions and simultaneous limited attention to environmental management could change considerably in response to disease reporting at the community level. In the Keiser et al. [108] study of co-infection noted in Table 6, it is important to note that clean water and sanitation, effectively maintained in these Cote d’Ivoire villages, would prevent the entire suite of intestinal parasitic diseases found to be present. Indeed, a focus on schistosomiasis or hookworm, for example – each on its own – makes no sense when considering disease control at the community level, and nor does siloed vector control.

Future research on global change and VBDs should give adequate attention to co-infection. Just the epidemiology and accompanying projections of climate change impacts would change considerably, as incorporation of disease interactions can lead to qualitatively different conclusions from what would be derived looking at a single disease at a time. Further, an important challenge to the epidemiological modeling community would be brought sharply into focus, as the literature on co-infection is very sparse and in great need of sustained development.

#### Taking a health systems approach

Addressing VBDs into the future demands that we take a health systems approach, in terms of strengthening existing initiatives, the ability to translate knowledge into action, and the capacity for organizations to promote community-based efforts. Rather than reinvent the wheel, our review (see Additional file 2) highlighted the continued marginalization of primary healthcare goals in countries and local settings where systems continue to be vastly inadequate [113]. In many respects, we know what to do – such goals have been reiterated many times. But underfunded health systems and ineffective health governance structures will likely continue to be major impediments to the successful control and future mitigation of VBD (see [114]).

Adapting to global change will require stronger primary healthcare systems and outreach mechanisms to deal with uncertain changes as they arise, such as epidemics and shifting transmission dynamics. Responding to atypical climatic variations that bring about Rift Valley Fever and malaria epidemics in East Africa require that the existing systems are in place to respond quickly when floods hit. Instituting vector control activities and community education quickly, as well as RVF vaccination for animals, is paramount to prevent deaths but cannot take place outside a strong existing institutional base [115]. The historical example of malaria control in China serves as a powerful example of the importance of country-ownership in promoting a health systems approach (see Table 7).

**Table 7:** Malaria control in China

China has had the longest running successful public health initiative focused on malaria of any country in the world. Starting gradually in 1950, systematically organized with a National Malaria Control Programme in 1955, and continuing to the present day, this adaptive, multiple-intervention, locally tuned effort at malaria suppression warrants in-depth examination and much more attention in contemporary discourse than it is receiving. Using 1949 as a starting point for baseline statistics, there were more than 30 million malaria cases in the country, and the mortality rate was approximately 1% per annum. Malaria was epidemic in 70–80% of all counties in the country, and represented 61.8% of the total recorded cases of acute infectious diseases in China in 1949. By the year 2000, there were 1.202 billion people living in areas where malaria incidence was less than 0.1 per thousand, and no county in the country reported an incidence above 10 cases per thousand. When the national control programme was initiated in 1955, it relied on primary health care networks as an organizational base, and made extensive use of community participation to respond to local needs. An intensive educational programme was put in place that featured advertising of integrated sets of interventions, giving balanced emphasis to both prevention and curative medicine. This balance has persisted to the present day. Indeed, successful suppression of malaria in the diverse Chinese ecosystems owes much to this holistic philosophy. Of special note is a particularly innovative use of intermittent irrigation for malaria control in Chinese ricefields, which was put into place in the 1970s, tuned to the local ecology of terraced ricefield systems The guiding framework for malaria control in China is the adaptive tuning of multiple interventions guided by performance-based ratings carried out over time. An additional form of monitoring also needs to be included here; namely, assessment of drug resistance. Not surprisingly, this phenomenon was, and continues to be, a challenging feature of antimalarial drug distribution in China. However, a research programme focused on drug resistance and the development of new drugs was initiated in response to this problem and plays an important role in the current version of the national programme. A central feature of the Chinese programme is that local ecology drove the choice of site-specific interventions. There was no imposition of general international guidelines about what interventions to emphasize globally. The only notion of scaling up that was brought into consideration was simply coverage of at-risk communities using tools appropriate to the local ecosystem. You could hardly have the intermittent irrigation strategy, so suitable for terraced rice fields, put into play in non-rice growing regions of China. A second key feature of this national programme is the organizational and communication infrastructure that facilitated a steady flow of information and local evaluations back and forth from the national programme to the village and district levels. The entire organizational structure of this programme provides an important role model for any country that is trying to implement a national malaria control effort. From Tang and Yang et al. [142, 143]	

Our review also highlighted the importance of building strong community-level outreach systems based around community workers and social services. Semenza [116] outlined a “lateral public health” approach to address adaptation needs in urban environments that incorporate social interventions (to advance bonding, bridging and linking social groups to enhance community capacity) and social service interventions (that integrate multiple sectors to reduce vulnerable population risks). Health system and social service interventions are the frontline in surveillance, diagnosis, treatment, education and wider community engagement. A central node in the network are community health workers who have local knowledge and access to community leaders that are invaluable for project managers both in terms of planning and implementation. But there is a need to scrutinize the performance of these workers by listening to them and learning from them, taking account of the contextual factors that influence their work and motivation [117]. Of course, a central tension is the widespread use of community volunteers to act as the main interface between programmes and communities. While in some contexts, this is certainly appropriate and effective, building strong community health systems require (modest) financial investment to support salaried outreach workers with appropriate monitoring support.

Health systems can normalize routines that become accepting of existing disease patterns and methods of organization and management; but considering global change requires tracking new patterns and addressing them in new ways. All of this demands investments in local public services, infrastructure, surveillance, outreach, and staff capacity. In increasingly decentralized government systems, the rhetoric is that costs need to be met by municipal and district funds, as well as national and international ones. However this is far easier said than done among populations most at risk of VBDs. Furthermore, local leadership in the health sector can be hampered by elites who do not reside in high-risk communities (and have little interest in their health), the propensity for seeking political capital instead of investing in long-term solutions and even the low credibility that some district and community leaders have with local people. Notions of citizenship and state responsibility influence VBD control; this is a central but poorly understood dimension of the health systems landscape that requires future research [118].

#### The architecture of participation

The architecture of factors that influence community participation also requires careful understanding, planning and monitoring. It is not enough to simply use the term “participation”, as this involves a gradient of ownership, inclusion/exclusion and empowerment [97]. Participation is a two-way street and should not be viewed as a way to cut costs by programme planners. It is rather the opposite, and requires its own type of capacity and financial investments [119]. Communities respond best when they see that civil authorities are taking responsibility for infrastructure issues, such as water provision and drainage problems. A major constraint for VBD control is that there are still relatively limited examples of how to institutionalize participatory approaches in developing countries in the context of limited resources and weak institutional support (see Table 8).

**Table 8:** Defining the architecture of community participation: the case of malaria

A systematic review on 60 years of research on malaria explored the architecture of community participation. The authors found and evaluated 60 academic papers that detailed how participation was implemented and commented on the strategies used and their effectiveness; however they found only 4 papers that explored efficacy in terms of disease impact. The authors highlighted 20 factors across the domains of individual, household, community and government/civil society that were found to play a major role in participatory approaches for malaria. These ranged from: i) disease perceptions, stigma, incentives and acceptability (individual-level); ii) gender and power relationships, cultural norms, access and geographical setting (household-level). It also included: iii) community characteristics and priorities, disease epidemiology, the complexity of the intervention, how communities are engaged and local priorities (community-level). Finally, (iv) it highlighted: the wider political environment, quality of the primary healthcare system, decentralization policy, advocacy and support, resources and other governance factors (government/civil society-level). The general conclusion of the review was that community participation continues to be marginalized in global efforts for malaria, despite its accepted benefits. A deficiency in the evidence-base for its effectiveness was noted, which generates problems for long-term funding and investment by global agencies with multiple, competing priorities. From Atkinson et al. [119]	

The capacity of local organizations and leadership need to be taken into account [120]. This includes an appreciation of power structures, inequalities at the local level and how gatekeepers may be silencing other community groups from having a voice, and participating. Generating a sense of ownership and collaboration takes time. Sequential meetings with stakeholders must be involved, including introducing the initiative, conducting situational analysis, allowing local perspectives to influence design and by discussing expectations. There is a need to understand the context of local organizations and facilitate learning so that they can help identify emergent problems as they come up.

In order for community-based interventions to be effective, adequate community awareness about the relationships between VBDs and social, environmental and climatic determinants are needed. This is likely to best occur in a participatory manner, which takes into account existing knowledge, practices, skills and priorities, while also recognizing the constraints posed by human behavior and structural conditions [121]. Operational and action-research approaches can certainly assist with the iterative process of moving pilot research projects into national campaigns [122, 123]; however, the example of the Chinese malaria programme noted above, and others, show what national ownership can accomplish at scale when driven by governments themselves.

#### Considering social difference

VBDs affect people and communities differently. Transmission includes significant social difference that is repeatedly highlighted by research on patterns of infection and the dynamics of community-based interventions. This includes attention to how livelihoods, gender, age, seasonal trends, socio-economic status, ethnicity and other factors create differential exposure and produce specifics types of vulnerabilities. Different social sub-groups also react differently to control strategies (See Table 9).

**Table 9:** Malaria and migrants in Cambodia

Population movement and forest-related activities and livelihoods along the Cambodia–Thailand border are major threats to the spread of artemisinin-resistant malaria. A mobile and migrant malaria plan was developed to target hard-to-reach populations as part of Cambodia’s National Malaria Elimination Strategy. Social science research was used to develop risk profiles of: seasonal, construction, mine and forest workers, as well as security personnel, visitors and cross-border travellers. Vulnerability scores were established based on knowledge, prevention measures, housing and immune/risk characteristics. A forest/malaria exposure index was created for each group. Lastly, an access to health services index was generated. These vulnerability, exposure and access indexes were then summarised into a matrix and used to identify groups of the highest risk. This has been used to tailor and target interventions to these very vulnerable social groups From Guyant et al. [59]	

An equity agenda emphasizes the most poor and marginalized, including migrants, ethnic minorities, women, children, and others [124]. Women make the majority of domestic decisions in the use of preventive measures and tend to the sick. Many community health groups, schoolteachers, primary health care providers, and traditional healers are also women. Education efforts often focus on women and children, since these groups are generally more receptive than men. Women tend to have larger local networks (or social capital) than men, such as mutual self-help groups and associations to access food, labor and cash. However they also tend to have more limited access to government outreach programmes (especially for agriculture), socio-economic development opportunities, and the ability to influence larger questions in the governance of resources [86]. This generates problems not only for women’s health, but also for their children. A large body of literature shows that children under 5 and pregnant women are at heightened risk of malaria and other VBDs. This underpins the logic of various initiatives aimed at identifying and managing these diseases in women and children [125].

Other groups, such as ethnic minorities, are equally vulnerable. In Panama, the Guna Amerindians recently experienced a severe malaria epidemic, driven largely by the El Niño Southern Oscillation, political instability, and health policy changes that ignored their needs [87]. Tribal communities worldwide, for example in India, have many barriers to VBD prevention, treatment and control that are geographic, cultural and social [126]. Spatial patterns and the practices of mobile and migrant populations are also key to VBD adaptation, as they can move pathogens into news areas within and between nations [127]. A study in Myanmar found that of mobile/migrant workers, only 15% were able to cite correct antimalarial drugs, and less than 10% believed that non-compliance with antimalarial treatment was a risk for drug resistance [128]. Hence, responding to social difference in VBD transmission and control will need to be a major aspect of mitigating future disease scenarios. But it is equally important that future programmes do not stigmatize these groups [129]. Migrants, indigenous groups, ethnic minorities and poor rural communities are already socially marginalized. As projects aim to strengthen their resilience and adaptive capacity, it is important to avoid using language that blames particular people as a source of infection and spread.

#### Use appropriate technology

Control strategies work best at the community-level when they take into account local perceptions of technology and how control tools are influenced by existing human behaviors and systems. Cultural norms and values are important to consider as they differ by region and social group, with implications for control tools (see Table 10). Just as social engagement strategies influence community responses to VBD interventions, local knowledge and perceptions of technology play a major role in mediating the level of acceptability and adaptability of programme tools, as field workers seek to influence uptake and engagement.

**Table 10:** Guppy fish and the control of dengue in Asia

Studies have shown that *Aedes aegypti* breeding sites are predominately in large water jars, tanks and drums in Cambodia and Laos. Distributing larvicidal fish into water containers in dengue-endemic areas could serve as a cultural acceptability tool due to preexisting aquaculture practices. A demonstration project funded by the Asian Development Bank (ADB) and WHO Western Pacific Region combined the use of a guppy fish distributing system, environmental control interventions and social mobilization to reduce *Aedes* larvae, pupae, and adult mosquito densities between 2009 and 2011. It used advocacy meetings, household visits, community meetings, advertising, mobile community outreach, drama, posters, school education, calendars and prizes. After 2 years, 80% of project households had guppies around their home. The success of the intervention was also clearly predicated on the socio-cultural acceptability of the technology. The project resulted in a decline in the number of water containers (jars, cement tanks, and drums) that were infested with *Aedes* larvae, which reduced from 40% to 3%. Furthermore, the project resulted in the successful establishment of a guppy breeding and distribution system at the national, provincial, and local levels in both countries, and generated multisectoral collaboration between ministries, nonprofit groups, schools, and health centers. However parallel research in Vietnam has showed the challenges of maintaining such a distribution system, and the importance of sustained government investment and support in order to provide oversight, sustained capacity building and guidance to local residents and grassroots NGOs as they attempt to maintain activities overtime [144]. From ADB and WHO [145]	

Using appropriate technology means paying attention to the ways in which communities use and perceive existing vector control strategies, as well as thinking about end-user preferences and concerns [130]. Values, norms and symbolic representations matter to how people use health technologies, and how they may modify them or use them in new ways. Many of the studies included in our review (see Additional file 2) highlighted the importance of considering different aspects of end-user adoption as programmes are implemented.

#### Integrated strategies and sustainable development

The history of VBD control – from malaria to sleeping sickness – teaches that reliance on one or two control tools is often ineffective and unsustainable. “Integration” is an important concept, but can mean many different things to different people in different contexts. Putting aside the more restrictive view of simply using two biomedical control tools together – like distributing bednets and providing anti-malarial drugs, for example – an integrated approach is about using knowledge of site specificities to tailor interventions. It is about an iterative and process-based way of solving these problems, and adapts to the social-ecological context, local livelihoods, political nuance and other factors.

Integrated vector management (IVM) has been widely recommended by the WHO and others, but continues to be greatly underdeveloped practically and in some ways theoretically. This is partially due to the challenges of funding, of inter-sectoral cooperation, of effective policy frameworks and of disciplinary divides [131]. But many historical antecedents do exist. Some were corporate sponsored control programmes that, aside from the current tendency to demonize corporations, were nonetheless considered far more effective than government programmes – for instance, Watson’s programmes in Northern Rhodesia and the Federated Malay States [132]. We can also learn lessons from indigenous systems that mitigate vectors and other pests. One of the most well-known examples of this is the traditional rice field irrigation system of the Balinese, which limited rice pests through water management and an intricate community system of land ownership and political organization [133].

The modern formulation of IVM has five key elements, all of which are relevant to efforts to address future VBD scenarios (Table 11). A study in Kenya found that sustaining IVM required strong community participation and support from multiple actors, such as community-based groups, NGOs, research institutes and different government departments [134]. Golding et al. [135] proposed that malaria, leishmaniasis, lymphatic filariasis and dengue are key candidates for an integrated vector control approach due to their geographic overlap and the effectiveness of ITNs and screens on all four, although no large-scale trial have been conducted. A good example of the narrow focus is the Global Programme to Eliminate Lymphatic Filariasis [112], which has essentially been focused on drugs, mostly neglecting to mentions that a common vector transmits both LF and malaria in the same communities.

**Table 11:** Key elements of integrated vector management (IVM)

1. Integration of chemical and non-chemical vector control methods 2. Evidence-based decision-making 3. Inter-sectoral collaboration 4. Advocacy and social mobilization 5. Capacity building From Beier et al. [111]	

A growing body of literature shows that socio-economic development drives major reductions in VBDs, not only in terms of improvements in the environment but also in terms of societal capacity to deal with future threats [51]. A long-standing effort to eliminate schistosomiasis in China has successfully used such an approach (see Table 12). High malaria transmission in Africa is often related to periods where vulnerable populations are stressed due to food insecurity, labor stresses, and where they have lack of access to healthcare; a study in Tanzania showed the importance of integrating malaria and food security programmes [98].

**Table 12:** An integrated approach to zoonotic schistosomiasis in the Dongting region of China

Control of *Schistosoma japonicum* in China has long been the focus of concerted state intervention over the past 60 years. The disease is predominated spread by cattle and water buffaloes. In the Dongting Lake region, changes to the vast marshland ecosystem and socio-demographic shifts continue to increase the range of the local snail host, while the completion of the Three Gorges Dam (the world’s largest hydroelectric project) is predicted to drive a re-emergence of the disease. While mass drug administration of praziquantel has been the mainstay of control in the Dongting Lake region, annual reinfection was found to occur in up to 20% of people due to occupational risks. To address this, an integrated approach was developed. This has included various educational strategies, such as videos and booklets delivered to schools. It has involved environmental modification, through building concrete irrigation systems and fences to separate livestock from water bodies. Campaigns for safe water and sanitation have been implemented, alongside focused snail control using niclosamide. Officials have also targeted high-risk groups for livelihood improvements and to reduce exposure time to infected water bodies. This has involved introducing mechanized farm equipment in an attempt to lower livestock herds as well as the resettlement of itinerant fishermen by providing free land and houses, which reduces the amount of time they spent in the lake. Compared to previous strategies, this integrated approach has significantly reduced the number of schistosome-endemic villages, populations at risk and the number of human cases between 1990 and 2010. From McManus et al. [146]	

Working towards sustainable VBD control in a changing world requires incorporating key principles of IVM and sustainable development: thinking holistically, understanding complex systems, using evidence to inform practice, working from an ecosystems perspective, promoting equity, thinking long-term, and being creative in the ways that interventions and initiatives promote integration. In this regard, the new Sustainable Development Goals (SDGs), with their focus on poverty alleviation, are certainly timely. There is also a need to question how different priorities between biosecurity and elimination-focused interventions interact with broader goals of strengthening primary healthcare, and how these may compete for funding and attention. While there are certainly synergies to be exploited between elimination efforts – currently supported by WHO and others for malaria, schistosomiasis, HAT, leishmaniasis and Chagas disease – it is important to emphasize that the trade-offs between these priorities need to be debated. Wider political economy issues – from corruption to the chain of dependence on aid funding and the international community – can be antithetical to burgeoning country-level efforts to engage in these prioritization debates for themselves. Furthermore, as elimination-targeted diseases become less common at community and country levels, prioritization becomes more difficult, even for globally-funded campaigns; hence integration between diseases and with wider health and development issues can actually work to the benefit of disease elimination.

#### Scaling-up: linking top, bottom and research

There is clearly a need to better synergize top-down and bottom-up approaches. The question is: at what scale can community-based approaches be successfully used and what is involved in scaling-up? Local adaptation does not occur in isolation from broader multilevel governance structures involving governments, donors, international agencies, NGOs and the private sectors. The risks of global change occur at larger scales and effective adaptation needs to involve institutional, infrastructural and governance changes at higher levels [66]. Too often, bottlenecks at the top of the health system mediate the performance of local interventions through maintaining institutional and systemic weaknesses.

A major challenge involves the favored emphasis on pilot studies, which are often research intensive but unsustainable and rarely integrated with the health system. The majority of papers cited in our review (see Additional file 2) described such demonstration projects in a small and localized setting. Pilot studies are certainly needed to experiment with new approaches and to generate data, but what happens after the research funding dries-up? Rarely does a research publication equate, by itself at least, to more effective on the ground disease control. Unfortunately, there is a major gap in the evidence, and in the types of systems needed to change this. Researchers tend to want to move their pilot interventions from small-scale, localized, well-funded and human resource-intensive demonstration studies to larger area-wide initiatives [136]. This may be the wrong way to go about things. Too often pilot projects occur in isolation. Rather, there is a fundamental need to move from research to adaptive implementation of tailored programmes, working within the existing health system and with other implementing partners. This allows for greater attention to scale and generalizability. Of course, scaling-up does not necessarily imply the transplantation of single interventions – it must be cognizant of the need to tailor approaches based on varying ecosystem and social dynamics.

There is a real danger, however, that centralized management systems, funding, oversight and institutional support will not allow for flexibility and tailored approaches on the ground [137]. Bureaucratic and institutional change needs to occur otherwise there is a risk of appropriation, or piecemeal application of a community-orientated approach (see Table 13). This is part of the logic and drive for pilot studies, which allow for more managerial control by a small group of highly trained professionals. Effective community-based interventions do not necessarily cost more than conventional approaches, but they do demand more time, new skill sets, iterative learning, and the transfer of decision-making power from experts to communities. This is very different to the ways in which most vector control departments, Ministries of Health and international organizations operate. Capacity building needs to occur at multiple levels. A process of ‘socialization’ and negotiation between funders, programme planners, field staff and community organizations can help overcome such problems.

**Table 13:** Scaling-up community empowerment in dengue control: the Cuban experience

A major challenge remains in scaling-up, financing, and institutionalizing community empowerment approaches to VBDs. In Cuba, dengue outbreaks helped facilitate the national *Aedes* control programme to include community empowerment as a major component of its national strategy. The existing programme involved a top-down structure, with over 30 000 field workers charged with entomological surveillance, larval source reduction, adult mosquito control, passive community education, and enforcement of household fines. Changes included a focus on bi-directional and experience-based learning, capacity building and shared leadership, with communities involved in decision-making and a greater focus on local-level organization. This included modifying existing guidelines and plans to incorporate participatory planning, behavioral research, training community leaders and community working groups and fostering intersectoral collaboration. An in-depth analysis over 5-years revealed a slow process of adoption of this new strategy, based on perceived matches between the needs of the national programme and how the empowerment approach could improve performance. The structure, practices, and organizational culture of the national control programme changed little. Important elements of the original empowerment strategy were left out. Major reasons included insufficient dissemination of the approach to government decision-makers, misinterpretation of empowerment principles, and a resistance to organizational change at the management level. This highlights the importance of properly conveying the benefits of an empowerment approach to decision-makers, and the need for better information exchange between those who develop participatory intervention designs, and national vector control staff. From Pérez et al. [147]	

Finally, there remains a continuing lack of evidence on how community-based approaches impact epidemiological trends and their cost-effectiveness, which is a key barrier to their wider scale diffusion. A major challenge is to enact mitigation efforts in the absence of epidemics and high numbers of human cases, which make prioritization by governments less likely. To truly scale-up, discussions of costs and benefits need to occur alongside mechanisms to better enact applied multidisciplinary research within existing national control policies and programmes. Real time operational research needs to be linked with decision-making.

## Conclusions

The importance of resilient global health systems to deal with vector-borne infections, and indeed most other human health threats, is truly a matter of life and death. There is little doubt that the spectrum of social, environmental and climatic changes occurring simultaneously in the twenty-first century will impact the distribution and incidence of VBDs. The specifics of how, where, when and why this will occur will vary greatly by disease, region, locality and social group. Uncertainties remain high, and current modeling efforts offer only limited applicability for policy design and programmatic orientation. In summary, vectors and pathogens change and adapt much quicker than scientific knowledge and, as history has shown, the systems of public health governance that preclude effective response.

While this generalization may be true, there are important pathways to strengthen resilience and adaptation to future VBD scenarios. Through concrete examples, this paper has emphasized the importance of taking a community-based approach and discussed a wide range of issues – from local knowledge, multidisciplinarity, integrated strategies, community participation, social difference, co-infection, and institutional dynamics – that should now be better incorporated. In light of the substantial commitments made at the Paris Agreement during the 2015 UN Climate Change Conference of the Parties (COP 21) and the renewed global movement to end poverty manifest in the Sustainable Development Goals (SDGs), now is the time to incorporate such an agenda within national adaptation policies and public health agendas. Doing so is paramount for the effective mitigation of future VBD spread in both urban and rural populations worldwide. Zika virus is stark reminder of this important need.

To address these challenges in a changing world, new forms of decision-making, partnerships, systems, and grassroots innovations are urgently needed. These need to account for the interrelationships between disease, natural systems and human institutions, politics, economics, behaviors and values. But adapting to future disease scenarios cannot take place without strengthening the existing public health infrastructure and addressing the social determinants of health. It is important that global change research, policy and practice for VBDs do not reinvent the wheel. Many validated prevention and control tools exist – from surveillance, chemical vector control, water, sanitation and hygiene (WASH), environmental modification, housing improvements, animal-based approaches and biomedical interventions (see our Additional file 2) – but lack sufficient political will and funding for scale-up. These are the ‘low-hanging fruit’ that need to be picked now in order to reap the full societal benefits down the road. These efforts should be targeted to hotspot areas at highest risk of the negative consequences of VBD change scenarios, which necessitates sound epidemiological science.

There are also important evidence gaps that should be filled, and more research funding is certainly needed. Greater attention to the importance of multidisciplinary research on shifting disease ecologies within the context of social-ecological systems should be one priority area. But by far more important is making research applicable to, and integrated within, existing national programmes. Bridging the gap between research and implementation is key in order to help design community-based interventions, facilitate their effective implementation, and scale-up. So too is the need to generate empirical evidence of their effectiveness, costs and sustainability. This is a major gap that likely impedes greater acceptance by national planners, policymakers and funders.

All of this will paradoxically require interventions be tailored to local community contexts and applied at large scales. Building local adaptive capacity will demand process-based, context-specific interventions but they must also be available for application across diverse contexts. Can local approaches be scaled-up meaningfully? While the answer is not simple, the scholarly literature reveals that attempts to do so have, to date at least, been few and far between. Global public health actors, as a community, need to do better. And we can. As we go about building the evidence-base, we need to better link research with policy and action [138]. We need to better use research in real-time to facilitate better implementation on the ground. Lessons learned should be rapidly translated into policy and practice, and success stories are vital to growing advocacy efforts. All of this will demand good management, sound biosocial science and strong leadership going forward into the future.

## Additional files

Additional file 1: Multilingual abstracts in the five official working languages of the United Nations. (PDF 591 kb)

Additional file 2: Community-based Approaches: What Works and Why? (DOCX 60 kb)

## Abbreviations

CBA: Community-based adaptation
CDC: Center for Disease Control and Prevention
HDSS: Health and Demographic Surveillance Systems
IPCC: Intergovernmental Panel on Climate Change
ITNs: Insecticide-treated bednets
IVM: Integrated vector management
LDCs: Least developed countries
LF: Lymphatic filariasis
LMICs: Low- and middle-income countries
NGO: Non-governmental organization
NTD: Neglected tropical disease
OECD: Organization for Economic Co-operation and Development
RVF: Rift Valley fever

## Footnotes

1 This includes an important initiative to understand the impact of climate change on human vulnerability to vector-borne diseases (malaria, schistosomiasis, African trypanosomiasis and Rift Valley fever) in sub-Saharan Africa. This project is funded by the International Development Research Centre (IDRC) and implemented by the Special Programme for Research and Training in Tropical Diseases (TDR). This review is funded by this project.

2 For an interesting historical example, see the venture “Industry and Tropical Health” run by the Harvard School of Public Health from 1950 to 1978.
