# Correction to: Addressing vulnerability, building resilience: community-based adaptation to vector-borne diseases in the context of global change

**DOI:** 10.1186/s40249-018-0387-6

**Published:** 2018-01-30

**Authors:** Kevin Louis Bardosh, Sadie J. Ryan, Kris Ebi, Susan Welburn, Burton Singer

**Affiliations:** 1 Department of Anthropology, University of Florida, Gainesville, USA; 2 Emerging Pathogens Institute, University of Florida, Gainesville, USA; 3 Department of Geography, University of Florida, Gainesville, USA; 4 Department of Global Health, University of Washington, Seattle, USA; 5 Center of Infectious Disease, University of Edinburgh, Edinburgh, UK

## Correction

After publication of this article [1] it came to our attention that the name of the author Sadie Ryan was incorrectly shown. Her correct name is Sadie J. Ryan. The original article has been updated to reflect this change.
