# Supplementary material for: Addressing vulnerability, building resilience: community-based adaptation to vector-borne diseases in the context of global change
Source: Infect Dis Poverty. 2017 Dec 11;6:166. doi: 10.1186/s40249-017-0375-2 (PMC5725972; doi:10.1186/s40249-017-0375-2)
Supplement: Supplementary file 1 — Multilingual abstracts in the five official working languages of the United Nations. (PDF 591 kb) [file 40249_2017_375_MOESM1_ESM.pdf]

Translation of the abstract into the five official working languages of the United Nations

معالجة الضعف، وبناء القدرة على التكيف: التكيف المجتمعي للأمراض المحمولة بالنواقل في سياق التغير العالمي

كيفن لويس باردوش، ريان سادي، كريس أبي، سوزان ويلبيرن، بيرتون سينجر

ملخص
خلفية: <bx/> إن الكوكب سريع التغير – إلى جانب التغيرات الاجتماعية والبيئية والمناخية – تشكل تحديات نظرية وعملية جديدة فيما يتعلق بالاستجابة للأمراض المحمولة بالنواقل. وهذه تشمل ديناميات التغير المكاني الزماني غير الخطية وعدم اليقين مرتبطة بالمناخ، والحيوانات، الأراضي والمياه، الغذاء، التسوية، الصراع، الإيكولوجيا والإنسان الاجتماعية والثقافية والاقتصادية والسياسي-المؤسسي المناظر الطبيعية. وحتى الآن، فقد هيمنت نمذجة الأمراض على الجهود البحثية، فلم يقدم الكثير من النصائح العملية لأصحاب القرار والممارسين الصحيين من أجل وضع السياسات والبرامج على أرض الواقع.
المحتوى الرئيسي: <bx/> في هذه الورقة، نقدم منظور حيوي-اجتماعي بديل يستند إلى رؤى العلوم الاجتماعية، وبالاستعانة بمفاهيم الضعف، والقدرة على التكيف، والمشاركة والتكيف المجتمعي. وقد استرشد تحليلنا باستعراض واقعي (مرفق في التذييل) يركز على سبعة مسببات رئيسية للأمراض المنقولة بالنواقل والمتأثرة بالمناخ: الملاريا، البلهارسيا، حمى الضنك، داء الليشمانيا، مرض النوم، داء شاغاس، حمى الوادي المتصدع. وهنا، حددنا تحليلنا للتدخلات المجتمعية السارية في سياق التغيرات العالمية ودراسات العلوم الاجتماعية الأوسع نطاقا. حيث نحدد ونناقش أفضل الممارسات والمبادئ المفاهيمية التي ينبغي الآن أن توجه الجهود المجتمعية المستقبلة للتخفيف من قابلية الإنسان للإصابة بالأمراض المنقولة بالنواقل. ونناقش أن هناك حاجة لتركيز الاهتمام أكثر ولاستثمارات في مجال المشاركة العامة الهادفة، والتكنولوجيات الملائمة، وتعزيز النظم الصحية، والتنمية المستدامة، والتغييرات المؤسسية الأوسع نطاقا والاهتمام بالمحددات الاجتماعية للصحة، بما في ذلك العوامل المؤدية إلى العدوى المشتركة.
الاستنتاج: من أجل الاستجابة بفعالية للسيناريوهات المستقبلية المحتملة للأمراض المحمولة بالنواقل في هذا العالم المتغير، فهناك حاجة للمزيد من الاهتمام في بناء نظم منصفة وقابلة للتكيف في الوقت الحاضر.

Translated from English version into Arabic by Suzan Alkhodair, through

TRANSLATORS
WITHOUT BORDERS

应对易感性，增强抵御能力:在全球化背景下以社区为基础应对媒传疾病

Kevin Louis Bardosh, Sadie Ryan, Kris Ebi, Susan Welburn, Burton Singer

摘要:
引言: 地球快速变化带来的威胁（如社会、环境和气候变化的耦合）对媒传疾病的防控提出了新的概念和挑战。如，与气候、动物、土地、水、食物、居住地、冲突、生态、人类社会文化、经济和政治制度相关的非线性和不确定的时空变化动态。迄今为止，以疾病建模为主导的研究工作为政策制定者和从业者在制定政策和项目规划时提供了确实可行的建议。
正文: 本文中，我们借鉴了易感性、抵御能力、参与度和基于社区适应的概念，提出了另一种基于社会科学的生物学观点。我们的分析以现实主义评论(见附录)的形式呈现，聚焦 7 种主要的对气候敏感的媒传疾病:疟疾、血吸虫病、登革热、利什曼病、昏睡病、恰加斯病和裂谷热。在全球化进程和众多社会科学文献的背景下，我们对现有社区干预措施进行分析，明确并讨论

了可指引未来社区行动的最佳做法和概念原则，以减轻人类对媒传疾病的易感性。我们认为以下几方面需要更多的关注和投资：有意义的公众参与、适宜技术、加强卫生系统、可持续发展、更广泛的体制变革以及关注健康的社会决定因素包括合并感染的驱动因素。
结论：为了有效应对不断变化的全球媒传疾病的未来发展趋势，目前需重视建立弹性和公平的系统。

Translated from English version into Chinese by Peng Song, edited by Pin Yang

CHINESE CENTER FOR DISEASE CONTROL AND PREVENTION
NATIONAL INSTITUTE OF PARASITIC DISEASES

Faire face à la vulnérabilité, renforcer la résilience : l'adaptation communautaire aux maladies à transmission vectorielle dans le contexte du changement planétaire

Kevin Louis Bardosh, Sadie Ryan, Kris Ebi, Susan Welburn, Burton Singer

Résumé

Contexte: Les menaces que représentent les évolutions rapides de la planète (changements sociaux, environnementaux et climatiques liés) posent de nouveaux défis conceptuels et pratiques dans la lutte contre les maladies à transmission vectorielle. Les difficultés sont liées notamment à la dynamique non linéaire et incertaine, dans le temps et l'espace, des changements associés au climat, à la faune, à la terre, à l'eau, à la nourriture, aux peuplements, aux conflits, à l'écologie et aux paysages socioculturels, économiques et politico-institutionnels. Les efforts de recherche sont dominés jusqu'à présent par la modélisation des maladies, dont la pertinence pratique pour les décideurs et les praticiens dans l'élaboration de politiques et de programmes sur le terrain s'avère limitée.
Corps du texte: Dans cet article, nous fournissons une perspective biosociale différente, fondée sur les connaissances en sciences sociales et s'appuyant sur les concepts de vulnérabilité, de résilience, de participation et d'adaptation communautaire. Notre analyse est éclairée par un examen réaliste (présenté en annexe) portant sur sept agents pathogènes majeurs sensibles au climat : paludisme, schistosomiase, dengue, leishmaniose, maladie du sommeil, maladie de Chagas et fièvre de la vallée du Rift. Nous nous concentrons, dans ce cadre, sur les interventions communautaires existantes dans le contexte des processus de changement planétaire et de la littérature en sciences sociales au sens large. Nous identifions et discutons les meilleures pratiques et les principes conceptuels qui pourraient à présent guider les efforts communautaires visant à atténuer la vulnérabilité humaine aux maladies à transmission vectorielle. Nous soutenons qu'une attention et des investissements plus ciblés sont nécessaires sur les questions de la participation pertinente du public, des technologies appropriées, du renforcement des systèmes de santé, du développement durable, des changements institutionnels à grande échelle et de l'attention portée aux déterminants sociaux de la santé, notamment aux facteurs de co-infection.
Conclusion: Afin de répondre efficacement aux scénarios futurs incertains suivant lesquels les maladies à transmission vectorielle pourraient évoluer dans un monde en mutation, il est nécessaire d'accorder dès à présent davantage d'attention à la construction de systèmes résilients et équitables.

Translated from English version into French by Emilie Rigault Fourcadier, through

TRANSLATORS
WITHOUT BORDERS

Преодоление уязвимости, повышение сопротивляемости: адаптация к трансмиссивным болезням на уровне общин в контексте глобальных изменений

Кевин Луи Бардош, Сэди Райан, Крис Эби, Сьюзан Уэлберн, Бертон Сингер

Аннотация

Справочная информация: Угроза стремительных изменений на планете – наряду с социальными, экологическими, а также климатическими изменениями – привносит в процесс реагирования на трансмиссивные заболевания новые концептуальные и практические проблемы. К ним относятся динамика нелинейных и неопределенных пространственно-временных изменений, связанная с климатом, с животными, с землёй, с водой, с питанием, с поселениями, с конфликтами, с экологией, а также с общечеловеческой, с общественно-культурной, с экономической и с политико-институционной ситуацией. На сегодняшний день в научных исследованиях преобладает моделирование болезни, которое обеспечивает политиков и практикующих специалистов довольно ограниченными практическими рекомендациями в области разработки мер и программ на местах.

Основной корпус:В этой статье предлагается альтернативная точка зрения, принимающая во внимание перспективу социальных наук, а также опирающаяся на концепции уязвимости, сопротивляемости, участия и адаптации на уровне общин. Данный анализ основан на реалистичном обзоре (приводится в приложении), включающем семь крупных патогенов трансмиссивных заболеваний, чувствительных к климатическим изменениям: малярию, шистосомоз, лихорадку денге, лейшманиоз, сонную болезнь, болезнь Шагаса, а также лихорадку Рифт-Валли. В анализе рассмотрены профилактические мероприятия на уровне общины, с проведением оценки в контексте процесса глобальных изменений и широкого охвата литературы по социальным наукам. В результате анализа был выявлен и изучен передовой опыт, а также основополагающие принципы, задействие которых необходимо для направления последующих усилий на уровне сообществ на снижение уязвимости людей перед трасмиссивными болезнями. В исследовании подчеркивается необходимость направления более пристального внимания и капиталовложений в следующие сферы: эффективное участие общественности, соответствующие технологии, укрепление систем здравоохранения, устойчивое развитие, более обширные институциональные изменения, а также внимание к социальным детерминантам здравоохранения, включая движущие силы коинфекции.

Заключение: Для того, чтобы эффективно реагировать в будущем на неопределённость развития трансмиссивных заболеваний в условиях меняющегося мира, большее внимание следует уделять созданию устойчивых и справедливых систем в настоящем.

Translated from English version into Russian by Liudmila Tomanek (nee Volynets), through

TRANSLATORS
WITHOUT BORDERS

Abordar la vulnerabilidad, reforzar la resiliencia: La adaptación comunitaria a enfermedades transmitidas por vectores en el contexto del cambio global

Kevin Louis Bardosh, Sadie Ryan, Kris Ebi, Susan Welburn, Burton Singer

Resumen

Contexto: La amenaza a un planeta que cambia rápidamente - combinado con el impacto de cambios sociales, ambientales y climáticos – plantean nuevos desafíos conceptuales y prácticos en respuesta a las enfermedades transmitidas por vectores. Esto incluye dinámicas de cambio espaciales y temporales no lineales e inciertas, asociados al clima, los animales, la tierra, el agua, los alimentos, los acuerdos, los conflictos, la ecología y panoramas socio-culturales, económicos y político-institucionales humanos. Hasta la fecha, los esfuerzos de investigación han sido dominados por la modelización de enfermedades, que le ha proporcionado un asesoramiento práctico limitado a las autoridades y profesionales en el desarrollo de políticas y programas en el campo.

Texto principal: En este trabajo, ofrecemos una perspectiva biosocial alternativa fundamentado en las ciencias sociales, basándose en conceptos de vulnerabilidad, resiliencia, participación y adaptación comunitaria. Nuestro análisis se basó en una revisión realista (presentada en el apéndice) centrada en siete patógenos importantes transmitidos por vectores: el paludismo, la esquistosomiasis, el dengue, la leishmaniosis, la enfermedad del sueño, la enfermedad de Chagas y la fiebre del Valle del Rift. Aquí situamos nuestro análisis de las intervenciones comunitarias existentes en el contexto de los procesos de cambio global y de la literatura de ciencias sociales en general. Identificamos y discutimos las mejores prácticas y principios conceptuales que ahora deben guiar los esfuerzos comunitarios futuros para mitigar la vulnerabilidad humana a las enfermedades transmitidas por vectores. Sostenemos que es necesario prestar más atención e invertir más en la participación significativa del público, las tecnologías apropiadas, el fortalecimiento de los sistemas de salud, el desarrollo sostenible, cambios institucionales más amplios y la atención a los determinantes sociales de la salud, incluidos las causas de infecciones conjuntas.

Conclusión: A fin de responder con eficacia a los escenarios futuros inciertos de las enfermedades transmitidas por vectores en un mundo cambiante, es necesario prestar más atención a la creación de sistemas resilientes y equitativos en el presente.

Translated from English version into Spanish by Reina X. Sanjurjo, through

TRANSLATORS
WITHOUT BORDERS
