# Supplementary material for: Addressing vulnerability, building resilience: community-based adaptation to vector-borne diseases in the context of global change
Source: Infect Dis Poverty. 2017 Dec 11;6:166. doi: 10.1186/s40249-017-0375-2 (PMC5725972; doi:10.1186/s40249-017-0375-2)
Supplement: Supplementary file 2 — Community-based Approaches: What Works and Why? (DOCX 60 kb) [file 40249_2017_375_MOESM2_ESM.docx]

**Additional file**

**Community-based Approaches: What Works and Why?**

Community-based interventions have proven to be effective in multiple contexts for the control and prevention of diseases of poverty [1-2]. However there is a major gap to guide best practice and policy options in the context of global change [3]. Without taking stock of already validated approaches and learning from them, it will be exceedingly difficult to imagine future pathways under the complexity of global change scenarios. Adaptation relies on innovation to grapple with a changing world, which will require working from, and modifying, existing tools.

Here we summarize the results of the systematic review on community-based interventions for 7 vector-borne diseases, relating past approaches to the context of future global change.^[[1]](#footnote-1)^ We develop a panoptic perspective on the types of approaches that are available and have been tested and evaluated. Furthermore, we ask: what works, why, in what context and for whom? Seven major types of community-based activities were identified, explored and analyzed. We situate these approaches within the broader context of socio-ecological systems theory and concepts of vulnerability and adaptation. The applicability of these approaches for contexts of global change is discussed, with specific examples provided from a country-level. This review informed the analysis and discussion in the attached paper, “*Addressing Vulnerability, Building Resilience: Community-based Adaptation to Vector-Borne Diseases in the Context of Global Change.”*

**1. Methodology of the Review**

We aimed to identify key community-based interventions and approaches that could help reduce vulnerability for 7 major VBDs in the context of global change. Due to the scarcity of scholarly literature that directly reports on global change, community-based adaptation and VBDs, there was a need to methodologically innovate and extrapolate. Hence the review involved multiple stages of data gathering, analysis and conceptual synthesis.

A search on both PubMed and Google Scholar databases found very little literature that documented community-based interventions to reduce global change risks for VBDs. Furthermore, very few scholarly articles were found that reported community-based studies on climate change and VBDs, aside from modeling efforts and some entomological studies.

**Table 2: Results of the Literature Review**

| **Disease** | **Number of articles identified** | **Number excluded** | **Number screened** | **Number included in qualitative synthesis** | **Number added to the database** |
| --- | --- | --- | --- | --- | --- |
| Chagas disease | 212 | 95 | 117 | 29 | 2 |
| Dengue | 295 | 248 | 47 | 55 | 8 |
| HAT | 163 | 126 | 37 | 25 | 8 |
| Leishmaniasis | 184 | 48 | 136 | 23 | 5 |
| Malaria | 2057 | 755 | 1302 | 419 | 9 |
| RVF | 22 | 15 | 7 | 5 | 2 |
| Schistosomiasis | 394 | 110 | 284 | 81 | 6 |
| **Total** | **3327** | **1397** | **1930** | **637** | **39** |

For these reasons, a systematic literature search was done to first synthesize major findings from existing community-based approaches to the 7 VBDs. The goal was to use this literature to identify the range of community-based mechanisms and pathways relevant to each VBD under social, environmental and climate change conditions. This search reviewed articles that described the monitoring and/or evaluation, either through qualitative or quantitative social research methods, of community-based prevention or control interventions for the 7 selected VBDs. Secondary searches were also done using Google Scholar to identify related articles, reviews and grey literature, such as project reports, that were then incorporated into the reviews as relevant. These sources were included if they provided additional conceptual insight, not all of which were disease specific.

For the systematic review, PubMed was used (see Table 2 above). This involved the following key search terms: [DISEASE X] and MONITORING or EVALUATION or QUALITATIVE or QUANTITATIVE or ETHNOGRAPHY or PARTICIPATORY and LOCAL or SOCIAL or PARTICIPATORY or INTERVENTION. Only articles published in English between 1990 and 2015 were included. In total, the search found 3,327 articles and included 673 articles in the qualitative synthesis. An additional 39 articles were included in the database – these were either cited in other studies or retrieved through our secondary literature search. Articles were excluded if they were not in English, not on the selected diseases, a review article or did not report the results of research activities that explored a community-based prevention or control intervention.

A total of 66% and 13% of the included articles were on malaria and schistosomiasis respectively. Not all articles that were included in the qualitative synthesis were included in this final report. A more selective approach was used, which relied on selecting articles that provided the greatest descriptive and analytical detail on vulnerability, the process of implementation, community responses and overall effectiveness factors. Using these selected articles, we followed a “realist review” approach, as outlined by Pawson et al. [4]. This focused on “providing an explanatory analysis aimed at discerning what works for whom, in what circumstances, in what respect and how.”

**2. Major Findings**

*2.1. Vector Surveillance and Risk Mapping*

One of the major needs in addressing future VBD scenarios relates to the uncertainties about the distribution and consequence of global change on vectors and pathogens. Surveillance systems remain the main source of information for policymakers and frontline health managers to assist with decision-making, resource allocation and response. Monitoring changes in atypical environmental conditions, such as rainfall and flooding in arid areas, and engaging in timely community-based vector control activities, have been shown to prevent epidemics of malaria and RVF [5]. To be effective, geographical information systems and surveillance must account for tracking and anticipating current and future conditions. This requires epidemiological and entomological evidence and investment in surveillance systems and modeling [6]. One tool that may prove to be particularly useful in this regard is the strengthening of existing local surveillance systems at the district level, particularly the use of existing Health and Demographic Surveillance Systems (HDSSs) that provide longitudinal data in established sites globally [7]. Such systems could guide ongoing adaptation and resilience efforts and the implementation of intervention packages.

| ***Box 1: Hunting Triatomine Bugs in Guatemala***  A number of initiatives have sought to involve communities in the surveillance and control of triatomine bugs in Latin America, the vectors of Chagas disease (Abad-Franch et al. 2011; Weeks et al. 2014). In Guatemala, a major Japan International Cooperation Agency (JICA) project (2000-2008) contributed to the interruption of *R. prolixus* transmission and significant reductions in *T. dimidiate.* This was achieved through surveillance, insecticide spraying and education [8].  But with decreasing funds for surveillance, a community-based bug-hunting campaign was piloted at scale. Using a participatory and intersectoral approach, ‘Chagas week’ encouraged community members to search their homes for Triatominae vectors*.* A range of stakeholders were involved (schoolchildren, community leaders, NGOs and community health volunteers) in outreach activities. The department of health showed strong ownership in the initiative, and also provided funds. Promotional flyers, bug reporting forms, lottery tickets and plastic bags for capturing the bugs were distributed, with the slogan: *Busque la chinche picuda y gane su premio* (Look for kissing bugs and win your prize).  The term “prize” was important, as bugs were exchanged with project officers for raffle tickets, which was especially popular in schools. The campaign was highly effective at improving vector surveillance, with a near 6-fold increase in the number of Triatominae vectors detected over more passive surveillance. This allowed for risk maps to be generated and for insecticide application to be used more effectively. It also raised political attention to the consequences of Chagas disease and the need to invest in sustained vector control.  From Yoshioka [9] |
| --- |

Additionally, despite the potential, community-based surveillance has not been widely used for most VBDs. Tsetse traps, triatomine “hunts”, and tracking and managing malaria cases are three notable exceptions. A study in Cambodia piloted the use of unsalaried village malaria workers to monitor potential artemisinin resistance in Plasmodium falciparum treated patients 72-hours after initiating treatment. The study found significant variation in the system depending on the level of supervision and training of village workers and health clinic staff [10]. These findings are repeated in the literature on tsetse traps, which highlight that communities are willing and able to deploy traps, monitor catches, maintain traps and liaison with public health and entomological authorities in the context of sufficient technical and financial support [11]. A study in Uganda explored community perceptions of traps in a control project, albeit the study is applicable to a surveillance context [12]. In villages where traps were familiar, people had very positive attitudes towards them, but where they had never been seen before they provoked fear and anxiety, related to witchcraft and “ghosts from the river.” While this highlights the importance of early engagement with communities and the need to consider local perceptions, surveillance incentives, didactic learning and a multi-sectoral approach are also all important components (see Box 1).

*2.2. Housing and the Domestic Environment*

Vector control includes a major focus on domestic spaces where human-vector-environment interactions take place. This ranges from bednets that keep sleeping quarters safe from nighttime mosquitoes, to the crevices in houses where triatomine bugs lay their eggs, to the water containers and recycling material where Aedes aegypt reproduce and spread dengue, CHIK and ZIKV. Numerous examples exist of simple housing improvements and changes in the urban environment that can reduce these diseases.

A large amount of research has been conducted on bednets, from local perceptions, usage, maintenance, efficacy and sustainable community-based distribution systems. Net care programs have been widely implemented using social and behavior change communication (SBCC) techniques. Studies have highlighted increased knowledge and attitudes to care and repair and modest increases to net durability– a study in Uganda showed a roughly 30% increase in net care, albeit with limitations for overall net condition [13]. Socio-economic status is often highlighted as a key influence on net condition, questioning how inequities reinforce an inability for poor households to care for their nets over time [14]. From the perspective of local people, nets can also catch fish, which can solve another problem for them: food insecurity.

A major issue for bednet success also relates to shifting sleeping patterns in response to notions of comfort (to avoid heat), livelihood patterns and nighttime socio-cultural and economic activities [15]. A study in Rwanda found that men were less likely to sleep under a bednet, even when they were available [16]. Perceptions of malaria risk are also important. Indifference can be rooted in a lack of fear of malaria infection, posing problems for elimination efforts [17]. An anthropological study on the implementation of long-lasting insecticidal nets and hammocks distributed to migratory Ra-glai ethnic minorities in Vietnam (who spend significant time in the forest during the malarial season) found that half of the local population did not use them in their forest plot huts, where they used slash and burn farming. This study recommended that programs target the agricultural fields where migrants seasonally reside, and not only permanent settlements that are more easily accessible to public health outreach teams [18]. In this sense, promoting community resilience involves maintaining and sustaining the goal of universal coverage by better targeting high-risk populations in remote regions. Despite a massive global campaign, insecticide-treated bednet (ITN) coverage is still not universal; an estimated 49% of the population at risk of malaria globally had access to ITNs in 2013, up from 4% in 2004. Questions about sustainable funding, as well as emerging resistance, evolutionary pressure on mosquito feeding behavior and possible long-term toxicity of pyrethroid insecticides, raise major concerns.

But not all vector control is about bednets or malaria. Community-based housing improvement interventions, for example, have been found to be very effective for Chagas disease, including a longstanding program in Venezuela [19]. Source reduction campaigns for Aedes mosquitoes have also been tested in multiple settings, especially where water usage and storage pattern are responsible for a large amount of Ae. aegypti larval habitats [20]. A frequent refrain from community-based research is that people lack knowledge on Aedes habitats and the importance of water containers; dengue is often associated with ‘dirty water’ and ‘worms’ [21].

| ***Box 2: A Community-based Aedes Source Reduction Campaign in Ecuador***  In recent years, dengue has replaced malaria as a major cause of febrile illness in parts of Ecuador. Mitchell-Foster et al. [22] conducted a randomized control trial of a participatory source reduction campaign in an urban area of Ecuador, where dengue epidemics are becoming more frequent due to climatic variation. The intervention was done in 10 intervention clusters, and consisted of an integrated dengue prevention approach using elementary school-based education and community-wide clean patio and safe container campaigns using neighborhood groups. This was found to have significantly reduced the pupa per person index, as compared to control clusters.  The intervention included practical skill development and application using a 6-week “Clean Patio and Safe Container” strategy, which mobilized students to remove discarded and unused containers from patios and cover water containers in peri-domestic spaces. The impact of this project was more significant in areas with long-standing community-based political action aimed at revitalizing government-community relations and infrastructure, such as community groups that had already mobilized for the installation and improvement of sewers, roads, lighting in public spaces, a children’s play-ground, increased police presence and improved garbage collection. The intervention also helped foster improvements in solid waste removal and public works reduction of standing water.  From Mitchell-Foster et al. [22] |
| --- |

Without a vaccine or effective treatment, and with significant gaps in the management of vertical dengue control programs [23], a number of randomized control trial cluster studies, using a participatory approach, have recently been done, with support from TDR, in Asia and Latin America, areas where global changes are shifting dengue dynamics. These have ranged from using schoolchildren and a backyard cleanup campaign in Brazil [24], integrated vector management and stakeholder engagement in Myanmar [25], women’s self-help groups and tailor-made water container covers using local carpenters in India [26], garbage collection services in Uruguay [27] and insecticide-treated net windows and water containers in Colombia [28]. All of these interventions, most of which are demonstration studies, reveal the need to maintain appropriate technology, motivate existing political support and address community mobilization (See Box 2). They show that participation was often lacking when no local community organizations existed, or where social instability and a lack of community cohesion were present, such as in migrant slum communities. While some have since been scaled-up across a much larger area, with the intention to understand how source reduction can impact dengue cases, publications are few and far between. A unique study in Cuba on the scale-up process showed that important elements of a participatory approach were left out due to insufficient dissemination of the approach to government decision-makers, misinterpretation of participatory principles, and a resistance to organizational change at the management level [29].

*2.3. Modifying Natural Environments*

Vectors are influenced by land use patterns, agriculture, water and human settlement. While insecticide application remains a mainstay of current control strategies, environmental modification has played a major historical role in vector control efforts [30], and still holds great potential today (see Box 3). Modifying river boundaries, draining swamps, clearing vegetation and modifying human habitation have all been used for malaria, schistosomiasis and sleeping sickness control. As efforts are scaled-up to eliminate VBDs, such as malaria, environmental modification has become more emphasized in the face of drug resistance, insecticide-resistant vectors and a shifting climate [31].

| ***Box 3: Cleaning Drains to Prevent Malaria in Dar es Salaam***  Community-based management of ecosystems offers a viable alternative to chemically-based vector control, but is under-utilized and under-studied. Between 2005 and 2007, researchers in Tanzania used community sensitization and mobilization to clean and manage two large drains (4 km long) as part of a pilot study. Drain cleaning activities were planned in consultation with community members and the city, municipal and ward leaders. Most of the hired workforce were local people, who cleaned the drains and did minor repairs. Community sensitization was done using seminars, mass meetings and house-to-house visits. The intervention reduced *Anopheline* breeding sites and cases of malaria. After 18-months, one of the drains was still been regularly maintained and cleaned, while the other had fallen into disrepair. The level of sensitization and community perceptions about the efficacy of the approach in reducing malaria influenced the management of the drains.  From Castro et al. [32] |
| --- |

Agricultural systems are also known to influence vector dynamics, and are effective routes for initiating community-based adaptations. A study in Kenya found that agricultural practices, such as dumping cassava tuber peelings, trench digging, irrigation and fishponds significantly favored mosquito breeding [33]. In Côte d'Ivoire, De Plaen et al. [34] showed how the intensification of irrigated rice cultivation lead to a reduction in the capacity of women to manage malaria disease episodes due to time constrains and increased labor demands. An intervention study in Morocco used participatory rural appraisal (PRA) to facilitate community analysis of schistosomiasis control options. Without outside support, a local irrigation committee repeatedly cleaned and removed vegetation from canals, which was found to be a very effective, low-cost and sustainable snail and schistosome control approach [35]. A local doctor was found to have played a key role in motivating the village committee, showing the important role of local champions.

People are willing to actively engage in their own effort to manage local environments (i.e. by managing stagnant water) but it is clear that communities require assistance to identify the most effective strategies to use, to facilitate collective action and to maintain activities over time [36].

*2.4. Animal-based Interventions*

Animal-based interventions involve engaging in the human norms, values and economics that govern our use of animals. One pathway for VBDs control involves the strengthening of livestock systems within a broader strategy for rural development. For example, the use of zooprophylaxis for the prevention of malaria in areas where livestock provide a protective layer, such as for Anopheles arabiensis in irrigated villages in Africa and Anopheles stephensi in India [37]. In other circumstances, however, closer contact with animals can increase chances for VBD infection, as with livestock slaughter practices in the transmission of RVF and dog-keeping practices for leishmaniasis. In Brazil, insecticide-treated collars have been used for leishmaniasis prevention. Mass dog culling has also been implemented, although the effectiveness of this approach has been widely questioned and has been resisted by local communities who find it inappropriate due to cultural values of dog ownership and companionship [38].

An integrated control strategy has been very effective in China for zoonotic schistosomiasis, which includes engaging farmers in the task of restricting cattle, who transmit schistosomes in their feces, from accessing snail-infested grasslands. This has involved using village committees to help introduce a system of mechanized agriculture to replace the need for large numbers of bulls, although the functioning of this system has not always worked as expected due to farmer interests and landscape factors [39]. Implementation of animal-based interventions also requires appreciating the ways that animals are valued, how livestock management is influenced by land patterns and climate, and the role of livestock in the rural economy. There may be opportunities to foster management changes that decrease natural resource challenges associated with increased VBD risk, and also help with broader livelihood improvements (see Box 4).

| ***Box 4: Strengthening Veterinary Services to Prevent Sleeping Sickness in Uganda***  In northern Uganda, an epidemic of sleeping sickness accompanied the end of war and conflict in the early 2000s. A public-private partnership was established to prevent human infection by implementing a series of mass cattle treatments using prophylactic drugs. For sustainability, a network of veterinary drug shops and community-based animal health workers were used to sell insecticides to farmers. This helped prevent animal and human trypanosomiasis as well as tick-borne cattle diseases. The veterinary network included community outreach and training following a social entrepreneurship model. While successful in some respects, community perceptions of drug and product efficacy, spray patterns, socio-economic trends and the incentives of community-based workers to treat cattle, generated a tension between business and sleeping sickness prevention. Other products only effective on ticks and not tsetse predominated in the market, and were often preferred by farmers due to lower up-front costs. Efforts to organize village-wide spray routines were not effective in the context of a post-conflict economy and a culture suspicious of state-led interventions. The lack of effective policy to treat cattle at livestock markets before they are moved to new districts continues to spread the human diseases to new areas.  From Bardosh [40] |
| --- |

*2.5. Water, Sanitation and Hygiene (WASH)*

The control of VBDs is frequently considered separate to the behavioral and infrastructural dimensions of water, sanitation and hygiene (WASH), despite a number of vector-borne infections having a major WASH dimension [41]. Sanitation and hygiene issues involve transferring infections from blood and feces to people, all of which have strong socio-cultural dynamics. Poorly constructed and maintained latrines, an essential component of WASH, can contribute to the breeding of *culex* mosquitoes, and so latrine maintenance can play a role in vector control.

Schistosomiasis is perhaps the best studied VBD in terms of community involvement with WASH. Transmitted through urination and defecation in waterways inhabited by the snail vector, the disease predominately infects young children and fishermen due to livelihood and recreational practices around rivers and streams [42]. Behavioral research has shown that water use and contact are complex, influenced by daily mobility patterns, livelihoods, geography and gendered use of space [43-44]. The staple of current schistosomiasis control is mass drug administration (MDA) of praziquantel; however research shows that reinfection is common and that drug resistance may be developing in certain contexts [45]. The current practice of deworming with no intervention to prevent re-worming amounts to installing an indefinite dependence on drugs for control that is not sustainable. An alternative, ‘water-based approach’ has been promoted, albeit mostly from the margins of mainstream policy [46]. The magnitude and pattern of investment required in WASH to address schistosomiasis, and other infectious diseases simultaneously, is difficult to gauge.

| ***Box 5: A Participatory WASH Approach to Prevent Schistosomiasis***  Schistosomiasis is widely endemic in Tanzania, with distributions predicted to change with climate change and other social and environmental shifts. Current control regimes focus almost exclusively on MDA, with little attention given to intensified health education and WASH. A participatory hygiene and sanitation transformation (PHAST) intervention was piloted in one hyper-endemic village in Ukerewe district. This approach facilitated a community group to develop an health action plan to improve WASH. A trainer-of-trainers workshop was used to organize a cadre of local volunteers to conduct classroom-based and village-based learning. This increased community knowledge and reduced water contact behavior in infected rivers, despite overall variations by sex, age-group and time of day. Children below the age of 15 years were most receptive to the PHAST intervention, while women did not change their water contact behaviors at all. After one-year, the intervention was found to not only have reduced overall risks but also decreased the wealth gap between households in the village by increasing household assets, housing and land ownership patterns. Community members attributed these changes to the participatory approach used and the secondary affects on community leadership and collective action.  From Mwanga et al. [47-48] |
| --- |

A participatory intervention in Nigeria controlled an outbreak of schistosomiasis by treating schoolchildren with MDA, rehabilitating water points and putting up health signs at the shores of endemic waterways [49]. However anthropological studies have shown that the banning of fishing and other livelihood activities in infected waters may not been very effective, and contributes to the stigmatization of poor communities that depend on fishing [42]. Results of a large-scale 25-year program to control Schistosoma mansoni in a Brazilian city showed a reduction from 70% to less than 2% [50]. This utilized an integrated approach combining mass chemotherapy with improvements in water supplies and sewage disposal in the context of widespread socioeconomic improvements. Although subsidized sanitation approaches have been found to be effective in schistosomiasis control [51], other studies have not shown an impact on infection rates [52]. Simply providing latrines may not be sufficient, as socio-cultural norms mediate their use. Participatory approaches to WASH have shown some results, and have currently been piloted in some schistosomiasis endemic areas (see Box 5). However it is important to note that such efforts need to put into place an ethos of maintaining engineering interventions – without this, WASH interventions cannot easily be sustained. Furthermore, little guidance is provided on the potential to scale-up: what would be the costs? The impacts? The programmatic structure and opportunities for integration? Further research is needed.

*2.6. Chemical Vector Control*

Government-funded vector control is central in the global effort to control vectors and pathogens. Despite calls for integrated vector management (IVM), the predominant focus of many efforts continues to be sporadic chemical application, with surveillance and education an irregular component. This includes blanket, and often reactive, use of larvicide, fogging, indoor residual spaying (IRS) and aerial spraying. More recent efforts include genetically modified mosquitoes, which present their own challenges of biological and social implementation, as many citizens are suspicious of Genetically Modified Organisms (GMOs) [53].

Most studies show major gaps in the operation of vector control programs – in staffing, capacity, management, funding and community engagement strategies [23]. Improving these capacities, and the ability to respond to future threats, is a major element of initiating successful community-based adaptation initiatives to tackle VBDs.

Two examples, from Burundi and in Latin America, are instructive. Research in the highlands of Burundi showed that the upper altitude limit for malaria was increasing and that a longer transmission season was occurring, which culminated in an epidemic in previously disease-free regions. Operational research was conducted to assess the effectiveness of using vector control, in this case IRS, in high-risk lowland (valley) zones to prevent further spread, which was found to be very effective [54]. This approach used highly technical teams and, like many studies on vector interventions, did not take a community-based approach, nor describe community dynamics that could have influenced effectiveness. However after the pilot studies funded by the research team, the vector interventions ceased because of a lack of funding, lack of integration within the primary healthcare context, and socio-political turmoil in the country.

A separate study in Guatemala, El Salvador, and Honduras explored the responsiveness of vector control teams between 2008 and 2012 to a novel community-based surveillance system that reported Triatoma dimidiate infestations, with the idea that government vector control officials would provide prompt IRS and educational advice on how to manage the infestation [8]. While community participation was very effective at detecting the vector, the responsiveness of the health system was lacking. The study explored how to reinforce vector control outreach by investigating 8 dimensions to implementation across 12 study areas, including: volume of vector notifications, local geography, demography, manpower, and managerial approach. They found that consistent performance monitoring within the local health system was the major pre-determining factor to effective responsiveness. These examples show the importance of considering the institutional context of implementation, and the need to build bridges between different stakeholders to make vector control efforts more receptive to community approaches. This is especially the case during the scale-up process (See Box 6).

| ***Box 6: Institutional Evolution of a Community-based Larval Source Reduction Initiative in Urban Tanzania***  Larval source management (LSM) campaigns require effective monitoring of larval sites. This requires fine spatial scales in urban landscapes. Participatory learning and mapping can play an important role, and can also assist project staff to be flexible to dynamic and emerging vector patterns. Gaining access to individual household plots in highly dense unplanned settlements is also a major problem that can be addressed by recruiting participants through local committees, who are familiar with the geography and with local residents. The quality of cartographic mapping plays a major role in planning, monitoring and managing larvicide application.  In the context of rapid urbanization in Tanzania, a citywide LSM campaign was implemented over a 14-year period in Dar es Salaam, Tanzania. This multi-sectoral community-based initiative used operational research to improve public health governance and sustainable service delivery for vector management. It used Community-Owned Resource Persons (CORPs), appointed through Street Health Committees, to visit every household plot weekly and apply larvicide (as needed) and survey potential mosquito larval habitats. Between 2004 and 2009, the program expanded to cover over 600,000 people. Managing such a project, with large numbers of irregularly paid volunteers, generated a number of frictions at different levels and between partners that had to be negotiated over time. The project found that large numbers of CORPS engaged by local leaders had poor performance and that there may be good reasons to hire smaller cadres that are better paid and incentivized.  One notable aspect of this project was that it ‘built upwards’, from neighborhoods to the city and national scale to influence policy and programming. Overtime, the City Council took greater responsibility for management from the research team, as planning was also decentralized to local administrative structures that enhanced community mobilization as well as funding support from the National Ministry of Health and Social Welfare. Research showed substantial reductions in malaria prevalence and mosquito density as larviciding was scaled-up. The project also successfully transitioned from a research initiative to a nationally-owned pubic health program. The collaboration between independent researchers and implementing partners allowed for a more honest assessment of performance and challenges.  From Chaki et al. [55] |
| --- |

*2.7. Access to Biomedical Interventions*

Not all attention to VBDs involves vectors, animals and environments but also diagnosis, treatment and mass chemotherapy approaches that target the pathogen. Most studies that explore community-based approaches to improved diagnosis and treatment focus on local illness perceptions, treatment seeking behavior, the system of healthcare and mass population-wide treatments.

Health messaging efforts aimed at conveying biomedical knowledge to local communities can become merged into pre-existing ideas and logics, called syncretic models. Like a game of ‘telephone’, they are interpreted according to the receiver’s presuppositions. Appreciating this dynamic can help make health promoters better adapt messages to local contexts [56]. Certain folk illnesses, that are considered to be 'malaria' by healthcare professionals, are categorized very differently by local people. A study on malaria in Tanzania found that homa ya malaria (in Swahili), or malaria fever, did not typically denoted the more severe forms of the disease, such as cerebral malaria in young children, severe anemia and malaria in pregnancy [57]. For these conditions, other sources of treatment are sought, such as from traditional doctors and herbalists.

In the context of malaria elimination and emerging drug resistance in Cambodia, adherence to treatment is important. Gryseels et al. [58] found three broad pathways for malaria treatment: i) the public sector; ii) the private sector; and iii) traditional treatments based on divination and ceremonial sacrifice. Even where good availability of anti-malarials existed in the public system, single-dose “cocktails” in the private sector were preferred due to notions of efficacy, local illness categories and socio-economic barriers that mediated patient choice. Local categorization and use of health technologies involve processes of transfer and appropriation embedded within a social context [59]. Barriers to the treatment of women for genital schistosomiasis in Egypt, for example, include a general neglect of women’s health, misconceptions about reproduction and limited access to formal health services for poor women [60]. A study in Suriname found that harmful non-biomedical substances, such as battery acid, lead, gasoline and insecticides, were regularly used to treat leishmaniasis [61]. Occupational contexts, such as working in gold and lumber sectors, meant that patients had access to these substances, while lower education, geographical distance to treatment centers, fear of injections, notions of masculinity and associative reasoning linking a ‘cruel disease’ to the need for a ‘cruel treatment’ were major drivers. Lastly, health-seeking behavior can also be influenced by ethnicity, as found in a study on new diagnostic tests for sleeping sickness in South Sudan [62].

Rapid decrease in vector-borne diseases has been reported following the implementation of well-organized community-based monitoring, training and support for village health workers [63], especially with the dissemination of Rapid Diagnostic Tests (RDTs) and arteminisin therapy (see Box 7). Studies have also assessed the uptake of training modules for rapid diagnostic tests, for example with private clinics, outreach workers and even teachers [62,64].

The population-wide delivery of chemotherapy continues to be a major component of the global fight against some VBDs, including mass drug administration (MDA) of praziquantel for schistosomiasis, and emerging support for focalized MDA for malaria elimination [65]. Research on MDA delivery channels of praziquantel has shown that a community-wide approach using community volunteers has the best coverage, compared to targeting only health facilities and schools [66]. Research has also shown that compliance with free treatments can be negatively effected by: a lack of information, rumors and mistrust, local understandings of disease, the method of drug distribution and population movement across borders [45]. Education of communities about the purpose of taking the drug is paramount, but often incompletely done. For example, after a 7-year campaign that combined MDA with health education in Senegal, only 30% of the population knew about the symptoms and mode of transmission, suggesting that education efforts were not being implemented with adequate attention to the implementation process and community perceptions [67]. There is a need to engage local leaders to take ownership of mass treatments by selecting volunteers and organizing the activities [68].

| ***Box 7: Malaria Control in a War Zone: The Case of Burma***  Active zones of war and conflict present major challenges for VBD control, and will continue to do so in the future. Large-scale malaria programs are rarely implemented in these contexts, especially as humanitarian agencies scale-back programs when violence erupts. In Eastern Burma, decades of conflict had displaced more than half-a-million people who lived in zones between rebels and government forces. A grassroots NGO operating health clinics and backpack teams in these areas implemented a network of village health workers to prevent malaria infection, which was estimated to account for 25% of overall morbidity and 40% mortality. This included training networks of village health teams, working under the guidance of clinic-based teams, so that they could distribute insecticide-treated nets, conduct simple diagnosis and provide malaria treatment. The intervention was scaled-up across more than 50 villages and was found to have reduced malaria infection rates. Regular ‘train-the-trainer’ workshops, held outside the conflict zone, were an important strategy to maintain health worker performance.  From Lee et al. [69] |
| --- |

When outbreaks of disease occur in new areas, communities adapt and change their perceptions and practices – Nazareth et al. [70] reported on changing practices after a dengue outbreak on a Portuguese island. But war and conflict present a different challenging situation for public health agencies (see Box 7). Humanitarian contexts make it hard to integrate screening and treatment of VBDs with the health system in areas experiencing epidemics during conflict (such as malaria in Afghanistan or sleeping sickness in the Democratic Republic of Congo) is complex since [71-72]. Targeted activities are needed in the absence of state infrastructure. Furthermore, resilience needs to be built across a gradient of social space to deal effectively with VBDs in conflict zones and post-conflict periods, where the transition from humanitarian programs to government and development agencies is fraught with social and political challenges.

**3. References**

1. Bardosh, K. (2014). Global aspirations, local realities: the role of social science research in controlling neglected tropical diseases. Infect. Dis. Poverty, 3(35), 10-1186.

2. Bhutta, Z. A., Sommerfeld, J., Lassi, Z. S., Salam, R. A., & Das, J. K. (2014). Global burden, distribution and interventions for the infectious diseases of poverty. Infect Dise of Pov, 3, 21.

3. Campbell-Lendrum, D., Manga, L., Bagayoko, M., & Sommerfeld, J. (2015). Climate change and vector-borne diseases: what are the implications for public health research and policy?. Philosophical Transactions of the Royal Society of London B: Biological Sciences, 370(1665), 20130552.

4. Pawson, R., Greenhalgh, T., Harvey, G., & Walshe, K. (2005). Realist review–a new method of systematic review designed for complex policy interventions. Journal of health services research & policy, 10 (suppl 1), 21-34.

5. Maes P, Harries AD, Van den Bergh R, Noor A, Snow RW, Tayler-Smith K, et al. (2014) Can Timely Vector Control Interventions Triggered by Atypical Environmental Conditions Prevent Malaria Epidemics? A Case-Study from Wajir County, Kenya. PLoS ONE 9(4): e92386. doi:10.1371/journal.pone.0092386

6. Hernández-Ávila, J. E., Rodríguez, M. H., Santos-Luna, R., Sánchez-Castañeda, V., Román-Pérez, S., Ríos-Salgado, V. H., & Salas-Sarmiento, J. A. (2013). Nation-wide, web-based, geographic information system for the integrated surveillance and control of dengue fever in Mexico. PloS one, 8(8), e70231.

7. Geubbels, E., Amri, S., Levira, F., Schellenberg, J., Masanja, H., & Nathan, R. (2015). Health & Demographic Surveillance System Profile: The Ifakara Rural and Urban Health and Demographic Surveillance System (Ifakara HDSS). International journal of epidemiology, dyv068.

8. Hashimoto, K., Zúniga, C., Romero, E., Morales, Z., & Maguire, J. H. (2015). Determinants of Health Service Responsiveness in Community-Based Vector Surveillance for Chagas Disease in Guatemala, El Salvador, and Honduras. PLoS Negl Trop Dis, 9(8), e0003974.

9. Yoshioka, K. (2013). Impact of a community-based bug-hunting campaign on Chagas disease control: a case study in the department of Jalapa. Memórias do Instituto Oswaldo Cruz, 108(2), 205-211.

10. Cox, J., Soley, L. D., Bunkea, T., Sovannaroth, S., Ty, K. S., et al. (2014). Evaluation of community-based systems for the surveillance of day three-positive Plasmodium falciparum cases in Western Cambodia. Malar J, 13(1), 282.

11. Brightwell, B., Dransfield, B., Maudlin, I., Stevenson, P., & Shaw, A. (2001). Reality vs. rhetoric–a survey and evaluation of tsetse control in East Africa. Agriculture and Human Values, 18(2), 219-233.

12. Kovacic V, Tirados I, Esterhuizen J, Mangwiro CTN, Torr SJ, Lehane MJ, et al. (2013) Community Acceptance of Tsetse Control Baits: A Qualitative Study in Arua District, North West Uganda. PLoS Negl Trop Dis 7(12): e2579. doi:10.1371/journal.pntd.0002579

13. Helinski, M. H., Namara, G., Koenker, H., Kilian, A., Hunter, G., Acosta, A., et al. (2015). Impact of a behaviour change communication programme on net durability in eastern Uganda. Malaria journal, 14(1), 366.

14. Panter-Brick, C., Clarke, S. E., Lomas, H., Pinder, M., & Lindsay, S. W. (2006). Culturally compelling strategies for behaviour change: a social ecology model and case study in malaria prevention. Social science & medicine, 62(11), 2810-2825.

15. Dunn, C. E., Le Mare, A., & Makungu, C. (2011). Malaria risk behaviours, socio-cultural practices and rural livelihoods in southern Tanzania: implications for bednet usage. Social Science & Medicine, 72(3), 408-417.

16. Kateera, F., Ingabire, C. M., Hakizimana, E., Rulisa, A., Karinda, P., Grobusch, M. P., et al. (2015). Long-lasting insecticidal net source, ownership and use in the context of universal coverage: a household survey in eastern Rwanda. Malaria journal, 14(1), 390.

17. Pulford, J., Oakiva, T., Angwin, A., Bryant, M., Mueller, I., & Hetzel, M. W. (2012). Indifferent to disease: A qualitative investigation of the reasons why some Papua New Guineans who own mosquito nets choose not to use them. Social Science & Medicine, 75(12), 2283-2290.

18. Peeters Grietens, K., Nguyen Xuan, X., Muela Ribera, J., Ngo Duc, T., van Bortel, W., Truong Ba, N., et al. (2012). Social determinants of long lasting insecticidal hammock-use among the Ra-Glai ethnic minority in Vietnam: implications for forest malaria control. PloS one, 7(1), e29991.

19. Briceño-León, R., & Méndez Galván, J. (2007). The social determinants of Chagas disease and the transformations of Latin America. Memórias do Instituto Oswaldo Cruz, 102, 109-112.

20. Seng, C. M., Setha, T., Nealon, J., & Socheat, D. (2009). Pupal sampling for Aedes aegypti (L.) surveillance and potential stratification of dengue high‐risk areas in Cambodia. Tropical Medicine & International Health, 14(10), 1233-1240.

21. Padmanabha, H., Soto, E., Mosquera, M., Lord, C. C., & Lounibos, L. P. (2010). Ecological links between water storage behaviors and Aedes aegypti production: implications for dengue vector control in variable climates. Ecohealth, 7(1), 78-90.

22. Mitchell-Foster, K., Ayala, E. B., Breilh, J., Spiegel, J., Wilches, A. A., Leon, T. O., & Delgado, J. A. (2015). Integrating participatory community mobilization processes to improve dengue prevention: an eco-bio-social scaling up of local success in Machala, Ecuador. Transactions of The Royal Society of Tropical Medicine and Hygiene, 109(2), 126-133.

23. Horstick, O., Runge-Ranzinger, S., Nathan, M. B., & Kroeger, A. (2010). Dengue vector-control services: how do they work? A systematic literature review and country case studies. Transactions of the Royal Society of Tropical Medicine and Hygiene, 104(6), 379-386.

24. Caprara, A., Lima, J. W. D. O., Peixoto, A. C. R., Motta, C. M. V., Nobre, J. M. S., Sommerfeld, J., & Kroeger, A. (2015). Entomological impact and social participation in dengue control: a cluster randomized trial in Fortaleza, Brazil. Transactions of The Royal Society of Tropical Medicine and Hygiene, 109(2), 99-105.

25. Wai, K. T., Htun, P. T., Oo, T., Myint, H., Lin, Z., Kroeger, A., et al. (2012). Community-centred eco-bio-social approach to control dengue vectors: an intervention study from Myanmar. Pathogens and global health, 106(8), 461-468.

26. Arunachalam, N., Tyagi, B. K., Samuel, M., Krishnamoorthi, R., Manavalan, R., Tewari, S. C., et al. (2012). Community-based control of Aedes aegypti by adoption of eco-health methods in Chennai City, India. Pathogens and global health, 106(8), 488-496.

27. Basso, C., da Rosa, E. G., Romero, S., González, C., Lairihoy, R., Roche, I., et al. (2015). Improved dengue fever prevention through innovative intervention methods in the city of Salto, Uruguay. Transactions of The Royal Society of Tropical Medicine and Hygiene, 109(2), 134-142.

28. García-Betancourt, T., González-Uribe, C., Quintero, J., & Carrasquilla, G. (2014). Ecobiosocial community intervention for improved Aedes aegypti control using water container covers to prevent dengue: lessons learned from Girardot Colombia. EcoHealth, 11(3), 434-438.

29. Pérez, D., Lefèvre, P., Castro, M., Toledo, M. E., Zamora, G., Bonet, M., & Van der Stuyft, P. (2013). Diffusion of community empowerment strategies for Aedes aegypti control in Cuba: a muddling through experience. Social science & medicine, 84, 44-52.

30. Keiser, J., Singer, B. H., & Utzinger, J. (2005). Reducing the burden of malaria in different eco-epidemiological settings with environmental management: a systematic review. The Lancet infectious diseases, 5(11), 695-708.

31. Beier, J. C., Keating, J., Githure, J. I., Macdonald, M. B., Impoinvil, D. E., & Novak, R. J. (2008). Integrated vector management for malaria control. Malaria journal, 7(1), S4.

32. Castro, M. C., Tsuruta, A., Kanamori, S., Kannady, K., & Mkude, S. (2009). Community-based environmental management for malaria control: evidence from a small-scale intervention in Dar es Salaam, Tanzania. Malaria journal, 8(1), 57.

33. Oladepo, O., Tona, G. O., Oshiname, F. O., & Titiloye, M. A. (2010). Malaria knowledge and agricultural practices that promote mosquito breeding in two rural farming communities in Oyo State, Nigeria. Malar. J, 9, 91.

34. De Plaen, R., Geneau, R., Teuscher, T., Koutoua, A., & Seka, M. L. (2003). Living in the paddies: a social science perspective on how inland valley irrigated rice cultivation affects malaria in northern Côte d'Ivoire. Tropical medicine & international health, 8(5), 459-470.

35. Boelee, E., & Laamrani, H. (2004). Environmental control of schistosomiasis through community participation in a Moroccan oasis. Tropical Medicine & International Health, 9(9), 997-1004.

36. Randell, H. F., Dickinson, K. L., Shayo, E. H., Mboera, L. E., & Kramer, R. A. (2010). Environmental management for malaria control: knowledge and practices in Mvomero, Tanzania. EcoHealth, 7(4), 507-516.

37. Mutero, C. M., Kabutha, C., Kimani, V., Kabuage, L., Gitau, G., Ssennyonga, J., et al. (2004). A transdisciplinary perspective on the links between malaria and agroecosystems in Kenya. Acta tropica, 89(2), 171-186.

38. Costa, C. (2011). How effective is dog culling in controlling zoonotic visceral leishmaniasis? A critical evaluation of the science, politics and ethics behind this public health policy. Revista da Sociedade Brasileira de Medicina Tropical, 44(2), 232-242.

39. Shi-Zhu, L., Ying-Jun, Q., Kun, Y., Qiang, W., Qiang, Z., Jun, L., et al. (2012). Successful outcome of an integrated strategy for the reduction of schistosomiasis transmission in an endemically complex area. Geospatial health, 6(2), 215-220.

40. Bardosh, K. (2016). Deadly Flies, Poor Profits and Veterinary Pharmaceuticals: Sustaining the Control of Sleeping Sickness in Uganda. Medical anthropology, 35(4), 338-352.

41. Pruss-Ustun, A. & Corvalan, C. (2006). Preventing disease through healthy environments: Towards an estimate of the environmental burden of disease. World Health Organization Press: Geneva.

42. Parker M, Allen T, Pearson G, Peach N, Flynn R, Rees N (2013) Border parasites: Schistosomiasis control among Uganda’s fisherfolk. J Eastern Afr Stud

6:97–122.

43. Hewlett, B. S., & Cline, B. L. (1997). Anthropological contributions to a community-based schistosomiasis control project in northern Cameroun. Tropical medicine & international health, 2(11), A25-36.

44. El-Katsha, S., & Watts, S. (2002). Gender, behavior, and health: schistosomiasis transmission and control in rural Egypt (No. 5377). American Univ in Cairo Press.

45. Parker, M., & Allen, T. (2011). Does mass drug administration for the integrated treatment of neglected tropical diseases really work? Assessing evidence for the control of schistosomiasis and soil-transmitted helminths in Uganda. Health research policy and systems, 9(1), 3.

46. Evan WS (2014). Water-based interventions for schistosomiasis control. Pathogens and global health, 108(5), 246-254.

47. Mwanga, J. R., Lwambo, N. J., Rumisha, S. F., Vounatsou, P., & Utzinger, J. (2013). Dynamics of people's socio-economic status in the face of schistosomiasis control interventions in Ukerewe district, Tanzania. Acta tropica, 128(2), 399-406.

48. Mwanga, J. R., & Lwambo, N. J. (2013). Pre-and post-intervention perceptions and water contact behaviour related to schistosomiasis in north-western Tanzania. Acta tropica, 128(2), 391-398.

49. Olaseha, I. O., & Sridhar, M. K. C. (2004). Participatory action research: community diagnosis and intervention in controlling urinary schistosomiasis in an urban community in Ibadan, Nigeria. International quarterly of community health education, 24(2), 153-160.

50. Sarvel AK, Oliveira ÁA, Silva AR, Lima ACL, Katz N (2011) Evaluation of a 25-Year-Program for the Control of Schistosomiasis Mansoni in an Endemic Area in Brazil. PLoS Negl Trop Dis 5(3): e990. doi:10.1371/journal.pntd.0000990

51. Pervilhac, C., Mshinda, H., Utzinger, J., Booth, M., & Tanner, M. (1997). Experiences with a multi-sectorial operation research programme for control of schistosomiasis in a Tanzanian district. African journal of health sciences, 5(3-4), 153-161.

52. Grimes, J. E., Croll, D., Harrison, W. E., Utzinger, J., Freeman, M. C., & Templeton, M. R. (2015). The role of water, sanitation, and hygiene in reducing schistosomiasis: a review. Parasit Vectors, 8, 766.

53. McNaughton, D. (2012). The importance of long-term social research in enabling participation and developing engagement strategies for new dengue control technologies. PLoS Negl Trop Dis, 6(8), e1785.

54. Protopopoff, N., Van Bortel, W., Marcotty, T., Van Herp, M., Maes, P., Baza, D., et al. (2008). Spatial targeted vector control is able to reduce malaria prevalence in the highlands of Burundi. The American journal of tropical medicine and hygiene, 79(1), 12-18.

55. Chaki, P. P., Kannady, K., Mtasiwa, D., Tanner, M., Mshinda, H., Kelly, A. H., & Killeen, G. F. (2014). Institutional evolution of a community-based programme for malaria control through larval source management in Dar es Salaam, United Republic of Tanzania. Malar J, 13(245), 10-1186.

56. Muela, S. H., Ribera, J. M., Mushi, A. K., & Tanner, M. (2002). Medical syncretism with reference to malaria in a Tanzanian community. Social Science & Medicine, 55(3), 403-413.

57. Winch, P. J., Makemba, A. M., Kamazima, S. R., Lurie, M., Lwihula, G. K., Premji, Z., et al. (1996). Local terminology for febrile illnesses in Bagamoyo District, Tanzania and its impact on the design of a community-based malaria control programme. Social science & medicine, 42(7), 1057-1067.

58. Gryseels, C., Grietens, K. P., Dierickx, S., Xuan, X. N., Uk, S., Bannister-Tyrrell, M., et al. (2015). High Mobility and Low Use of Malaria Preventive Measures Among the Jarai Male Youth Along the Cambodia–Vietnam Border. The American journal of tropical medicine and hygiene, 93(4), 810-818.

59. Granado, S., Manderson, L., Obrist, B., & Tanner, M. (2011). Appropriating “malaria”: local responses to malaria treatment and prevention in Abidjan, Cote d'ivoire. Medical anthropology, 30(1), 102-121.

60. Talaat, M., Watts, S., Mekheimar, S., Ali, H. F., & Hamed, H. (2004). The social context of reproductive health in an Egyptian hamlet: a pilot study to identify female genital schistosomiasis. Social science & medicine, 58(3), 515-524.

61. Ramdas, S. (2012). Cruel disease, cruel medicine: self-treatment of cutaneous leishmaniasis with harmful chemical substances in Suriname. Social Science & Medicine, 75(6), 1097-1105.

62. Palmer, J. J., Surur, E. I., Checchi, F., Ahmad, F., Ackom, F. K., & Whitty, C. J. (2014). A Mixed Methods Study of a Health Worker Training Intervention to Increase Syndromic Referral for Gambiense Human African Trypanosomiasis in South Sudan. PLoS neglected tropical diseases, 8(3), e2742.

63. Huda, M. M., Hirve, S., Siddiqui, N. A., Malaviya, P., Banjara, M. R., Das, P., ... & Mondal, D. (2012). Active case detection in national visceral leishmaniasis elimination programs in Bangladesh, India, and Nepal: feasibility, performance and costs. BMC public health, 12(1), 1001.

64. Witek-McManus, S., Mathanga, D. P., Verney, A., Mtali, A., Ali, D., Sande, J., et al. (2015). Design, implementation and evaluation of a training programme for school teachers in the use of malaria rapid diagnostic tests as part of a basic first aid kit in southern Malawi. BMC public health, 15(1), 904.

65. Silumbe, K., Chiyende, E., Finn, T. P., Desmond, M., Puta, C., Hamainza, B., et al. (2015). A qualitative study of perceptions of a mass test and treat campaign in Southern Zambia and potential barriers to effectiveness. Malaria journal, 14(1), 171.

66. Mafe, M. A., Appelt, B., Adewale, B., Idowu, E. T., Akinwale, O. P., Adeneye, A. K., et al. (2005). Effectiveness of different approaches to mass delivery of praziquantel among school-aged children in rural communities in Nigeria. Acta tropica, 93(2), 181-190.

67. Sow, S., Vlas, S. J., Mbaye, A., Polman, K., & Gryseels, B. (2003). Low awareness of intestinal schistosomiasis in northern Senegal after 7 years of health education as part of intense control and research activities. Tropical Medicine & International Health, 8(8), 744-749.

68. Massa, K., Magnussen, P., Sheshe, A., Ntakamulenga, R., Ndawi, B., & Olsen, A. (2009). Community perceptions on the community-directed treatment and school-based approaches for the control of schistosomiasis and soil-transmitted helminthiasis among school-age children in Lushoto District, Tanzania. Journal of biosocial science, 41(01), 89-105.

69. Lee, C. I., Smith, L. S., Shwe Oo, E. K., Scharschmidt, B. C., Whichard, E., Kler, T., et al. (2009). Internally displaced human resources for health: villager health worker partnerships to scale up a malaria control programme in active conflict areas of eastern Burma. Global public health, 4(3), 229-241.

70. Nazareth, T., Sousa, C. A., Porto, G., Gonçalves, L., Seixas, G., Antunes, L., et al. (2015). Impact of a Dengue Outbreak Experience in the Preventive Perceptions of the Community from a Temperate Region: Madeira Island, Portugal. PLoS neglected tropical diseases, 9(3), e0003395.

71. Tong, J., Valverde, O., Mahoudeau, C., Yun, O., & Chappuis, F. (2011). Challenges of controlling sleeping sickness in areas of violent conflict: experience in the Democratic Republic of Congo. Confl Health, 5(7).

72. Kevany, S., Sahak, O., Workneh, N. G., & Saeedzai, S. A. (2014). Global health diplomacy investments in Afghanistan: Adaptations and outcomes of global fund malaria programs. Medicine, Conflict and Survival, 30(1), 37-55.

1. It is important to note that taking such a “disease specific approach” does not mean that we are neglecting the important issue of co-infection, solely emphasizing so-called “vertical approaches” or overlooking the importance of integrated strategies – far from it. [↑](#footnote-ref-1)
